# Separating the effects of temperature and absorbed X-ray dose on unit-cell volume

**DOI:** 10.1107/S2059798326008612

**Published:** 2026-09-15

**Authors:** Robert J. Southworth-Davies, James W. Murray, Ian Carmichael, Enrique Rudiño-Piñera, Martin Weik, Elspeth F. Garman

**Affiliations:** a Department of Biochemistry University of Oxford Dorothy Crowfoot Hodgkin Building, South Parks Road OxfordOX1 3QU United Kingdom; b Life Sciences Imperial College Exhibition Road LondonSW7 2AZ United Kingdom; c Notre Dame Radiation Laboratory University of Notre Dame Notre Dame IN46556 USA; d Laboratorio de Bioquímica Estructural, Instituto de Biotecnología Universidad Nacional Autónoma de México Cuernavaca 62271Morelos Mexico; e Université Grenoble Alpes, CEA, CNRS, Institut de Biologie Structurale 38044Grenoble France; University of Western Australia, Crawley, Australia

**Keywords:** unit-cell volume, X-ray radiation damage, macromolecular crystallography, atomic *B* factors

## Abstract

The behaviour of the unit-cell volume of crystals of the iron-storage molecule ferritin, both in the apo and the holo form, and of influenza A virus subtype N9 neuraminidase at 100 K and over a controlled cryo-temperature series was investigated. The purpose of this study was to separate out the effects of dose and temperature on the protein by assessing the changes in the unit-cell volume. Over the wide range of X-ray doses examined in this work, the irreversible effect of dose on the unit-cell volume could be distinguished from the reversible (below 160 K) temperature-induced effects. Specific structural damage effects were not reversible.

## Abstract 2

The nature of the expansion of the crystal unit-cell volume with absorbed dose and with temperature was examined for three different proteins.

## 1. Introduction

The irradiation of protein crystals with highly intense X-ray synchrotron radiation at cryo-temperatures (100 K) results in an increase in the unit-cell volume (Yonath *et al.*, 1998 ▸; Burmeister, 2000 ▸; Ravelli & McSweeney, 2000 ▸; Teng & Moffat, 2000 ▸; Weik, Kryger *et al.*, 2001 ▸; Weik, Ravelli *et al.*, 2001 ▸; Murray & Garman, 2002 ▸; Ravelli *et al.*, 2002 ▸; Sliz *et al.*, 2003 ▸; Liebschner *et al.*, 2015 ▸ and many subsequent observations). This is a permanent change, increasing with the absorbed dose, and at the flux densities available from third-generation synchrotrons is thought not to be due to any temperature increase of the protein crystal caused by X-ray beam heating (Kriminski *et al.*, 2002 ▸; Kuzay *et al.*, 2001 ▸; Nicholson *et al.*, 2001 ▸; Sliz *et al.*, 2003 ▸; Snell *et al.*, 2005 ▸; Mhaisekar *et al.*, 2005 ▸). A series of measurements using four different temperature-ramping regimes were made by Ravelli *et al.* (2002 ▸) on crystals of holoferritin which contain one Fe atom for every two amino acids. In one of the protocols, the X-ray beam was turned off for 15 min between data-collection sweeps at the different temperatures, allowing the crystal temperature to re-equilibrate and any beam-induced heating to dissipate. In this case the unit-cell volume did not revert as would have been expected in the absence of structure-altering radiation damage, suggesting that the accumulated dose had resulted in a permanent change to the structure and/or crystal packing, as discussed more generally in Warkentin *et al.* (2013 ▸).

At 100 K, the change in unit-cell volume with absorbed dose has been observed to be linear up to 10 MGy for hen egg-white lysozyme (HEWL; Teng & Moffat, 2000 ▸), 2.7 MGy for holoferritin (Ravelli *et al.*, 2002 ▸) and 31 MGy in the case of myrosinase (Burmeister, 2000 ▸). The Teng & Moffat (2000 ▸) study noted that above 10 MGy the unit-cell volume expansion becomes nonlinear and starts to plateau. A similar observation was made by Sliz *et al.* (2003 ▸), where there was some levelling off of the unit-cell volume increase, measured against frame number, with a flux density above 3.3 × 10^17^ photons s^−1^ mm^−2^ at a third-generation synchrotron, although no dose estimations were included in that study. Thus, the unit-cell expansion rate can be seen to be highly material-dependent.

The reason for the expansion of the unit-cell volume with absorbed dose may be due to hydrogen gas (arising from the radiation-induced hydrogen abstraction from organic molecules; Meents *et al.*, 2010 ▸) accumulating at grain boundaries and crystal imperfections. Burmeister (2000 ▸) suggested that it could arise either from an expansion of the solvent part of the protein crystal, or from an expansion of the protein, or both. A reason for the expansion of the solvent could be the loss of hydrogen bonds owing to the transformation of water molecules into radical species such as the hydroxyl radical as radiation damage progresses. Molecular H_2_ (Meents *et al.*, 2010 ▸) and perhaps a small amount of CO_2_ (Sage *et al.*, 2011 ▸) (from decarboxylation of Asp and Glu residues) may accumulate in interstitial spaces, causing a build-up of internal pressure. Gas bubbling is commonly observed in cryo-electron microscopy experiments on grids and was reported to be molecular H_2_ (Leapman & Sun, 1995 ▸).

There has also been a report that at room temperature (RT; 292 K) the unit-cell volume can contract with absorbed dose (Boutet & Robinson, 2006 ▸). Boutet and Robinson found that when microcrystals of holoferritin were exposed to X-rays from an undulator beamline at the Advanced Photon Source (APS), Chicago at RT, a sudden lattice contraction was observed following a characteristic latent period (corresponding to an absorbed dose of approximately 20 MGy), ultimately leading to collapse of the crystal lattice. A contraction of the unit-cell volume with dose was observed by Ravelli *et al.* (2002 ▸) at the highest temperature tested in those experiments (180 K). A more recent study suggesting a convenient method of obtaining RT datasets following cryo data collection reported that the expansion of the unit-cell volume for samples warmed from 100 K to RT was around 4%, with the exception of one sample cooled after treatment with Paratone-N oil, which shrank by 12% (Huang *et al.*, 2024 ▸).

Although, as mentioned above, unit-cell volume increases due to accumulated dose are not thought to be a result of temperature rises, recent measurements at the European Synchrotron Radiation Facility Extremely Brilliant Source (ESRF–EBS) in Grenoble have shown that using the flux densities now available at fourth-generation synchrotrons, heating of RT samples of metals and cerium oxide can lead to temperature increases of as much as 400 K. Since temperature rises also cause expansion of the crystal lattice parameters, these were used as a metric to calculate the temperature rises from the known thermal expansion properties of the samples (Lawrence Bright *et al.*, 2021 ▸). For RT protein crystals, a very recent comprehensive modelling exercise calculated the predicted temperature increase in simulated data for beams of various sizes (1–20 µm) and average crystal composition over various exposure times in beams ranging from 1 × 10^11^ to 1 × 10^15^ photons s^−1^. The authors concluded that using a flux of 1 × 10^15^ photons s^−1^ and 20 µs exposures, 1 and 5 µm crystals would suffer temperature increases of 180 and 32 K, respectively (Appleby *et al.*, 2026 ▸). These are significant rises, and experimental experience with these new very high flux-density beams will allow the heating models to be refined.

For cryocooled macromolecular protein crystals, it is known that increasing the temperature can cause an expansion of the unit-cell volume (Müller *et al.*, 2002 ▸; Tilton *et al.*, 1992 ▸; Weik, Kryger *et al.*, 2001 ▸). The study by Tilton *et al.* (1992 ▸) on RNAase crystals held at temperatures between 98 and 320 K showed that the protein expanded by 0.4% per 100 K, and that the expansion corresponded to a rearrangement of the protein molecules within the unit cell. Using a sealed-tube X-ray source, Weik, Kryger *et al.* (2001 ▸) determined the change in relative unit-cell volume of trigonal crystals of *Torpedo californica* acetylcholinesterase (*Tc*AChE) when the temperature was raised from 100 to 180 K to be +3.7%. This large change was due to water within the large solvent channels forming crystalline ice above the solvent glass-transition temperature (155 K) in these crystals. Based on earlier experiments on trigonal *Tc*AChE crystals at the ESRF on beamline ID14-4 (Ravelli & McSweeney, 2000 ▸), it was estimated that the dose-dependent increase at 100 K in the experiment by Weik, Kryger *et al.* (2001 ▸) on a sealed-tube source would be 0.0001%. If one assumes the dose-dependent increase of the unit-cell volume to be temperature-independent, the 3.7% expansion of the unit-cell volume must thus have been dominated by the temperature rise to 180 K. Further experiments on a home source (little or no radiation-damage effects) showed that ramping orthorhombic (16 × 21 Å solvent channels, ∼42% solvent content) and trigonal (12 × 16 Å narrower solvent channels, ∼18% solvent) trypsin crystals from 100 to 200 K gave linear increases in unit-cell volume but that an abrupt nonreversible decrease was observed at 185 and 195 K in the two systems, respectively, as the temperature was increased, with concomitant ice rings appearing in the diffraction patterns (Weik *et al.*, 2005 ▸).

Müller *et al.* (2002 ▸) measured changes in *d*-spacings (the spacing between the lattice planes) at a beam energy of 12.4 keV (wavelength 1.000 Å) for organic compounds as a function of temperature from 100 to 220 K and found that the rate of change of *d*-spacing with time (d*d*/d*t*) depended strongly on the temperature. In the first experiment, the thermal expansion of the compounds was determined using a low-intensity (33% transmission) beam. In further experiments, the irreversible changes in *d*-spacing were investigated in detail at high (100% transmission) photon flux densities of 4.0 × 10^12^ photons s^−1^ mm^−2^. The small-molecule light-atom compounds investigated showed a linear increase of the lattice spacing with absorbed radiation dose up to X-ray flux densities of ∼5.0 × 10^12^ photons s^−1^ mm^−2^ [estimated dose rate 1.4 kGy s^−1^, calculated with the original *RADDOSE* program (Paithankar *et al.*, 2009 ▸) using the values given by Müller and coworkers]. The rate of the lattice-spacing change with temperature was also linear within this range of flux densities. It was possible to separate a putative temperature-induced change in *d*-spacing from that related to dose because of this linear behaviour. Within the accuracy of their method (∼1–2 K) no significant increase in temperature of the sample due to beam heating could be observed up to a flux density of ∼5 × 10^12^ photons s^−1^ mm^−2^. A recent study was aimed at deconvoluting the radiation-induced lattice response from temperature-induced effects in two mercury-containing and one bismuth-containing small-molecule crystals. The observed expansion with temperature and with dose was compared with the calculated thermal expansivities coupled with second-rank tensor analysis to separate the effects and then to obtain a measure of the radiation-damage susceptibility of the materials (McMonagle *et al.*, 2024 ▸).

During an investigation of the dependence of specific radiation damage (breakage of disulfide bridges, decarboxylation of Asp and Glu) on temperature, Warkentin and coworkers measured the unit-cell expansion of thaumatin crystals with X-ray dose at five different temperatures between 25 and 300 K. They found a linear relationship between the unit-cell volume and the molecular radius of gyration, with the volume increasing with dose at all of the temperatures tested (Warkentin *et al.*, 2012 ▸).

Flash-cooling protein crystals from RT to 100 K causes a protein crystal unit-cell volume to contract substantially, reducing it by 2–7% (Juers & Matthews, 2001 ▸). This could be caused by a combination of contraction of the protein molecule itself and by rearrangement of the crystal, or by contraction of the solvent, or both. Crystals at 100 K had unit-cell volumes about 6% smaller than those at RT, whereas the protein molecule itself contracts by only 1–2%. This led Juers & Matthews (2001 ▸) to consider repacking as the source of the unit-cell contraction observed in a study of *Escherichia coli* β-galactosidase cooled from 293 to 100 K, with the driving force being entropic in nature. This allows the protein to adopt different conformations but to maintain its overall shape, accommodating the need of the protein to be flexible and so that it is able to occupy a wide part of the phase space of the physical system. Further work (Juers *et al.*, 2018 ▸) investigated the contraction with temperature of protein crystals having 32–67% solvent content and concluded that the solvent contracted 2–11 times more than the protein for the cases tested (Juers *et al.*, 2018 ▸).

An obvious question is why data might be collected at temperatures other than 100 K or RT (292 K). Measurements at a range of temperatures are necessary in studies involving enzyme kinetics, mechanisms and protein dynamics because of the differing stability of reaction intermediates at different temperatures (Weik, Ravelli *et al.*, 2001 ▸; Keedy *et al.*, 2015 ▸; Keedy, 2019 ▸). It is therefore important to be aware of the effect of temperature change on the unit-cell volume, and thus the effect on the experiment in terms of possible non-isomorphism. When carrying out experiments involving protein dynamics over a range of temperatures, the behaviour and phase transitions of the solvent over the temperature range in which the protein is being studied must also be considered. Previous work has probed how the relative unit-cell volume varies with absorbed dose at temperatures above and below the solvent glass transition at 155 K (Weik, Kryger *et al.*, 2001 ▸; Weik, Ravelli *et al.*, 2001 ▸). In the absence of the crystallization of solvent water, the unit-cell volume changes were observed to be fully reversible (low-dose experiment, approximately 10^4^ Gy), indicating that they were a consequence of thermal expansion of the solvent and/or protein molecules (Weik, Kryger *et al.*, 2001 ▸). However, if crystallization of the solvent occurred, the unit-cell volume increase was large, was irreversible and was dominated by the difference in volume between the amorphous and the crystalline form of the solvent. It should also be noted that the magnitude of the unit-cell volume increase is different even between crystals of the same size and type at the same temperature, thus excluding it as a metric for monitoring the rate of radiation damage (Murray & Garman, 2002 ▸; Ravelli *et al.*, 2002 ▸). Weik, Ravelli *et al.* (2001 ▸) found that the relative increase in unit-cell volume of *Tc*AChE with dose was larger at 100 K (0.4%) than at 155 K (0.2%), showing that radiation-induced increases in the unit-cell volume did not follow the degree of structural damage to particular amino acids, which was greater at 155 K. The mechanism leading to unit-cell volume increase and the mechanism leading to specific damage were therefore distinct. The researchers suggested that the ‘unit-cell volume increase mechanism’ was caused mainly by primary damage, whereas the ‘mechanism leading to specific damage’ was caused mainly by secondary damage. At 100 K, radicals created in the solvent area are trapped and their creation increases the unit-cell volume linearly with dose. At 155 K, above the solvent glass transition, radicals are able to diffuse and recombine, which could, along with possible hydrogen-gas diffusion, explain the observed nonlinear unit-cell volume increase at higher temperatures. This is in accord with the observation that 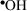 radicals produced at lower temperatures can become mobile upon raising the temperature to 110 K (Owen *et al.*, 2012 ▸). The increased mobility of 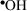 radicals and thus their greater ability to attach to solvent-exposed amino acids at 160 K compared with at 100 K was also postulated (along with an increase in static disorder) to account for the structural differences in experiments with thermolysin crystals. It was found that at 160 K more residue types were affected (Lys, Asp, Gln, Pro, Thr, Met and Asn) than at 100 K (Met, Asp, Glu and Lys) (Juers & Weik, 2011 ▸).

In the work described here, the behaviour of the unit-cell volume of crystals of the iron-storage molecule ferritin with and without a phosphate–iron mineral core and of influenza A virus subtype N9 neuraminidase, over a controlled temperature series between 100 and 165 K, was investigated. The purpose of these experiments was to separate the effects of dose and temperature on the protein crystals by examining the changes in the unit-cell volume.

The structure of cubic apoferritin has been deposited in the PDB as entry 1ier (Granier *et al.*, 1997 ▸) at a resolution of 2.26 Å. Ferritins are a family of iron-storage proteins that are found in animals, plants, fungi and bacteria. Horse spleen ferritin is a four-helix bundle protein of 174 amino-acid residues, which forms a 24-mer of molecular weight 440 kDa with 432 cubic symmetry. The 24 symmetrically related subunits form a near-spherical hollow shell with an 80 Å internal diameter (Ford *et al.*, 1984 ▸), and in the crystal four ferritin 24-mers are contained within a single unit cell. These 24-mers are used in a physiological context to store around 1760 Fe atoms (Owen *et al.*, 2006 ▸) in the core of the molecule. In the crystal, they form a face-centred cubic lattice, space group *F*432, with a cell length of approximately 181 Å and a solvent content of 62%.

The large unit cell of ferritin gives many reflections per diffraction image and this, combined with the high (cubic) symmetry, allows relatively easy unit-cell determination, since only a single cell-edge length requires fitting. It was therefore less likely (than for noncubic systems) that errors in the unit cell would compensate for errors in other geometrical and experimental parameters, such as the sample-to-detector distance. The easy availability of ferritin and the relatively good diffraction (2.1 Å resolution in the case of apoferritin on beamline ID14-4 at the ESRF) properties of the crystals make this an excellent test protein crystal for radiation-damage studies. The metal ions dramatically increase the X-ray absorption cross-section of holoferritin and thus greatly enhance its sensitivity to irradiation (Ravelli *et al.*, 2002 ▸; Owen *et al.*, 2006 ▸). Apoferritin contains no iron in its core and has a similar X-ray absorption coefficient (0.462 mm^−1^; 12.7 keV; values calculated using *RADDOSE*; Murray *et al.*, 2004 ▸; Paithankar *et al.*, 2009 ▸) to typical metal-free protein crystals (for example HEWL, absorption coefficient of 0.272 mm^−1^at 12.7 keV). The iron-containing holoferritin is a suitable comparison since the crystals are isomorphous to those of apoferritin but they have a much higher X-ray absorption coefficient (1.277 mm^−1^ at 12.7 keV). The difference in iron-core content allows one to sample a protein whose forms are at the two extremes of metal-ion content, apoferritin being devoid of metal ions and holoferritin having approximately only two amino acids per Fe atom.

Neuraminidase subtype N9 was also chosen as a test protein crystal for these experiments as, like ferritin, it has a cubic space group (*I*432) and large unit-cell dimensions (*a* = *b* = *c* ≃ 181 Å). Also, similarly to ferritin, the molecules are arranged in a near-spherical manner.

The results of our experiments on these three proteins show that there is no interdependent relationship of the unit-cell volume between dose and temperature over the wide range of doses and temperatures examined here, and the irreversible effect of dose could clearly be distinguished from the reversible temperature-induced effects.

## 2. Materials and methods

### 2.1. Crystallization

Ferritin originating from horse spleen was obtained from Sigma, Poole, UK (product codes: apoferritin, A-3641; holoferritin, F-4503) as filtered solutions (apoferritin 55 mg ml^−1^, holoferritin 85 mg ml^−1^) in NaCl and diluted to 20 mg ml^−1^ with 0.1 *M* NaCl. It was crystallized at a concentration of 20 mg ml^−1^ by hanging-drop vapour diffusion using a mother liquor consisting of 0.6 *M* ammonium sulfate, 10 m*M* cadmium sulfate. A drop produced by mixing 4 µl protein solution and 4 µl well solution on a cover slip was placed above a 400 µl volume of the mother liquor in the well. The crystals grew at 292 K to approximately 100 µm in the *x*, *y* and *z* dimensions and were cryoprotected by replacing 40% of the water in the well solution with glycerol.

Neuraminidase subtype N9 (A/Tern/Australia/G70c/75) was purified and crystallized using previously established procedures (Laver *et al.*, 1984 ▸). Crystals were grown by hanging-drop vapour diffusion against a reservoir consisting of 1.9 *M* potassium phosphate pH 6.8, starting with equal volumes of N9 neuraminidase solution (10–15 mg ml^−1^ in water) and potassium phosphate buffer (1.4 *M* KH_2_PO_4_:3 *M* K_2_HPO_4_ in an 8:4 ratio) pH 6.6 at 20°C. Large rhombic dodecahedral crystals in space group *I*432 with *a* = *b* = *c* ≃ 181 Å (maximum dimensions exceeding 0.5 mm) grew within a few days. The crystals required a cryo-buffer with 40%(*v*/*v*) glycerol in the mother liquor for cryoprotection (again they were cryoprotected in solutions where the water in the mother liquor was replaced by different percentages of glycerol) and the *in situ* sequential soaking procedure detailed in Garman (1999 ▸) was employed. Cryo-buffer containing 10, 20, 30 and then 40%(*v*/*v*) glycerol in the mother liquor was each delivered twice, mixed, and then withdrawn over a total period of 5 min.

### 2.2. Data collection

Data were collected from two crystals of horse spleen holoferritin on beamline ID23-1 (13.2 keV/0.939 Å) at the ESRF: ‘holo constant series 1’ (HCS_1) at 100 K and, under a varying temperature regime, ‘holo temperature series 1’ (HTS_1). The maximum value considered safe when varying the temperature was 165 K. This avoided crystalline ice formation which would have led to irreversible changes to the cell dimensions (Weik, Kryger *et al.*, 2001 ▸). Since crystalline ice formation produces characteristic and easily identifiable ice rings on a diffraction pattern, the images were monitored to ensure that the sample mounted in the X-ray beam remained free of crystalline ice. It was not possible to calculate the absorbed dose directly for either holoferritin crystal as beamline ID23-1 at that time did not allow the integrated counts measured by the inline pin diode to be recorded. The collection regimes were therefore matched as closely as possible with each other, *i.e.n* consecutive data collections with identical detector distance, oscillation angle, exposure time, number of images and datasets. Thus, for HCS_1 and HTS_1 dose estimates were later inferred from the behaviour of the second holoferritin constant-dose series collected on ESRF beamline ID14-4 (see below), for which doses could be estimated from the beamline parameters.

Data were collected from four crystals of horse spleen apoferritin, two crystals of holoferritin and two crystals of N9 neuraminidase on beamline ID14-4 (12.7 keV) at the ESRF. All of the ‘constant series’ (CS) data were collected at 100 K, whereas the ‘temperature series’ (TS) data were collected under varying temperature regimes. The datasets of ‘apo constant series 1’ (ACS_1) were separated by a series of ‘burns’ where the crystal was exposed to the unattenuated beam. This caused extensive radiation damage to the crystal and meant that the total dose absorbed by this crystal was much larger (97 MGy) than the dose for the other apoferritin crystals which had few or no burns between datasets. The ‘holo constant series 2’ (HCS_2) and ‘holo temperature series 2’ (HTS_2) were subjected to burns but not between every dataset. The N9 constant series (N9CS_1) and ‘N9 temperature series’ (N9TS_1) both consisted of sequential datasets without any burns.

Beam calibration was carried out as described previously (Owen *et al.*, 2009 ▸). The photon flux was then input into *RADDOSE* (Murray *et al.*, 2004 ▸; Paithankar *et al.*, 2009 ▸) for all dose calculations. The *RADDOSE* input parameters for the composition of ferritin were taken from Owen *et al.* (2006 ▸), where a full description of the measurements and composition calculations for both holo and apo ferritin is given. These values were measured by particle-induced X-ray emission, microPIXE (Garman & Grime, 2005 ▸), on both liquid protein and crystal samples. It was found from a comprehensive series of measurements that there were on average 25.2 S atoms per monomer, 73.6 Fe atoms per monomer and 2.3 Cd atoms per monomer for holoferritin crystals, and 58.7 S atoms per monomer, 0 Fe atoms per monomer and 5.6 Cd atoms per monomer for apoferritin crystals. The non-integer values are due to the CdSO_4_ in the crystallization buffer, and the stoichiometric results have errors of <10%. The dose values for HTS_1 and HCS_1 were inferred from the change in Wilson *B* factors, *B*_rel_ = *B*_*n*_ − *B*_1_ (Kmetko *et al.*, 2006 ▸), during HCS_2 in the following manner. The HCS_2 *B*_rel_ values were plotted against the dose values obtained from *RADDOSE* and a linear fit (*R*^2^ = 0.9931) allowed a ‘coefficient of sensitivity’, *S*_AD_ = Δ*B*_rel_ /8π^2^Δ*D* of 0.007 Å MGy^−1^ to be extracted. Since crystals of the same protein have been found to have similar values of *S*_AD_ at cryotemperatures (Kmetko *et al.*, 2006 ▸), this value was used to estimate the doses for HTS_1 and HCS_1, despite it being rather lower than that observed, for instance, for hen egg-white lysozyme. It should be noted that the original *RADDOSE* code output the maximum dose in the 21-voxel array into which the crystal was partitioned for the calculation.

**Figure 1:**
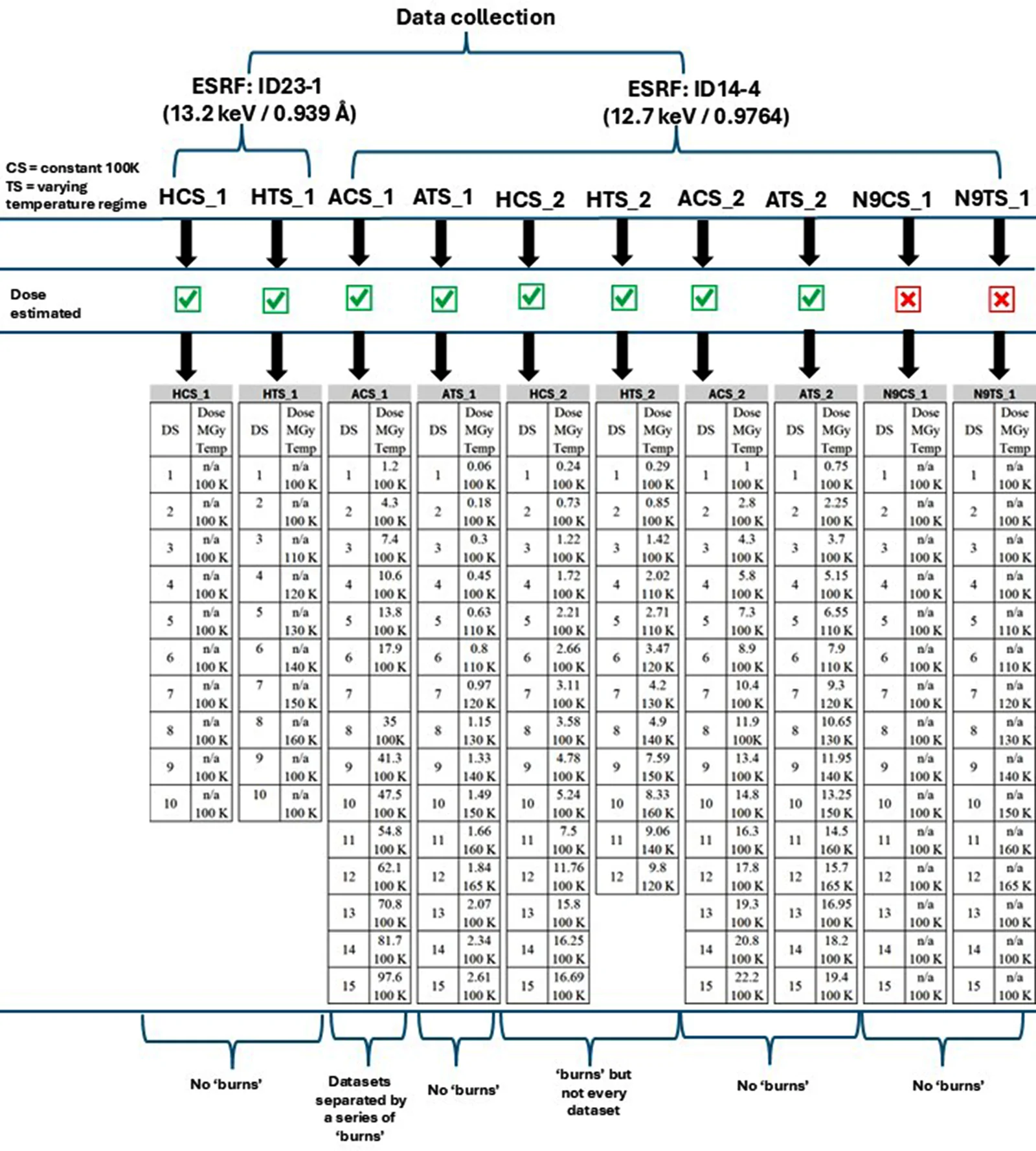
Summary of all of the datasets collected.

Experimental parameters for each crystal are shown in Table 1 ▸ and all of the datasets described below are summarized in Fig. 1 ▸.

### 2.3. Data processing

All datasets were processed using the *CCP*4 program suite (Agirre *et al.*, 2023 ▸). Using *MOSFLM* (Leslie, 2006 ▸), they were integrated between 40 and 2.1 Å resolution for the ACS_1, HCS_1, ATS_1 and HTS_1 data, between 40 and 2.3 Å resolution for the ACS_2, HCS_2, ATS_2 and HTS_2 data, and between 40 and 2.1 Å resolution for the N9 neuraminidase data.

*MOSFLM* was used to refine the sole unit-cell parameter of the first dataset (DS1), allowing the sample-to-detector distance to vary. This distance was then fixed for all subsequent refinements of the integration parameters. Minute instrumental and systematic errors give rise to small deviations between the predicted and observed spot positions, and those are partly compensated for by the refinement of the detector parameters. When the detector parameters are fixed, these errors are subsumed by changes in the refined crystal parameters, such as unit-cell volume. The extracted unit-cell lengths are however strongly dependent on the processing protocol used and the way in which the data-reduction package is applied, so exactly the same processing protocol was necessary for each of the datasets. The unit-cell values for dataset 2 onwards in each series were therefore extracted from *MOSFLM* with the sample-to-detector distance remaining fixed.

*SCALA* (Evans, 2006 ▸) was used to scale together multiple observations of the same reflection, merge multiple observations into an average intensity for a dataset and assign identical test reflections (for the *R*_free_ set) for every dataset in the series. *TRUNCATE* (French & Wilson, 1978 ▸) was employed to convert recorded intensities into structure-factor amplitudes.

The output files from *SCALA* were combined together using *CAD* and the datasets from one crystal series were scaled with respect to each other using Wilson scaling in *SCALEIT* (Howell & Smith, 1992 ▸) to extract the isotropic *B *factor for calculation of the sequential differences, *B*_rel_ (Kmetko *et al.*, 2011 ▸), between datasets relative to the first dataset.

Since the starting PDB model of ferritin (PDB entry 1ier; Granier *et al.*, 1997 ▸) was derived from a crystal in the same space group and had the same unit-cell parameters as the crystals used in these experiments, a molecular-replacement step was unnecessary. For the ATS_1, HCS_1 and HTS_2 datasets, *REFMAC*5 (Murshudov *et al.*, 1999 ▸) was used for rigid-body refinement of the model (stripped of all nonprotein atoms) against the first dataset for each crystal and *ARP*/*wARP* (Perrakis *et al.*, 1997 ▸) was used to add water molecules to the models. Water placement and removal were iterated with the maximum-likelihood refinement procedure of *REFMAC*, as directed by *ARP*/*wARP*. Restrained refinement was completed using *REFMAC* iterated with manual model building using *Coot* (Emsley *et al.*, 2010 ▸). The various model-analysis tools available in *Coot*, such as the Ramachandran plot of φ and ψ angles along the backbone, were used to monitor the quality of the refined structure. The density-fit analyses in *Coot* of the 2*mF*_o_ − *F*_o_ and *F*_o_ − *F*_o_ maps against residue number were examined as well as the rotamer analysis of each of the residues. This information was used to manually alter the model so that the residues sat in the allowed areas of the Ramachandran plot, the density of the 2*F*_o_ − *F*_o_ map was well fitted and the number of unusual rotamers was at a minimum. This iterative procedure continued until the crystallographic *R* value was <0.22 and *R*_free_ was <0.26.

*CAD* was then used to combine the phases from the refined model of DS1 for each crystal with the structure factors of the sequential datasets from that crystal using the same *R*_free_ set and *FFT* was used to produce *F*_o1_ − *F*_o*x*_ Fourier difference maps between datasets (where *x* is any dataset other than 1). The Fourier difference maps therefore combined the phases derived from the refined model of DS1 of each series with the measured structure-factor amplitudes of each sequential dataset. The maps were very clear and showed damage to glutamate, aspartate and methionine residues, which are the classic sites of radiation damage (Burmeister, 2000 ▸; Ravelli & McSweeney, 2000 ▸; Weik *et al.*, 2000 ▸). Note that there are no disulfide bonds in ferritin. All peaks in electron-density difference maps were compared for structures derived from data of the same resolution.

## 3. Results

### 3.1. Changes in unit-cell volume with dose and temperature

**Figure 2:**
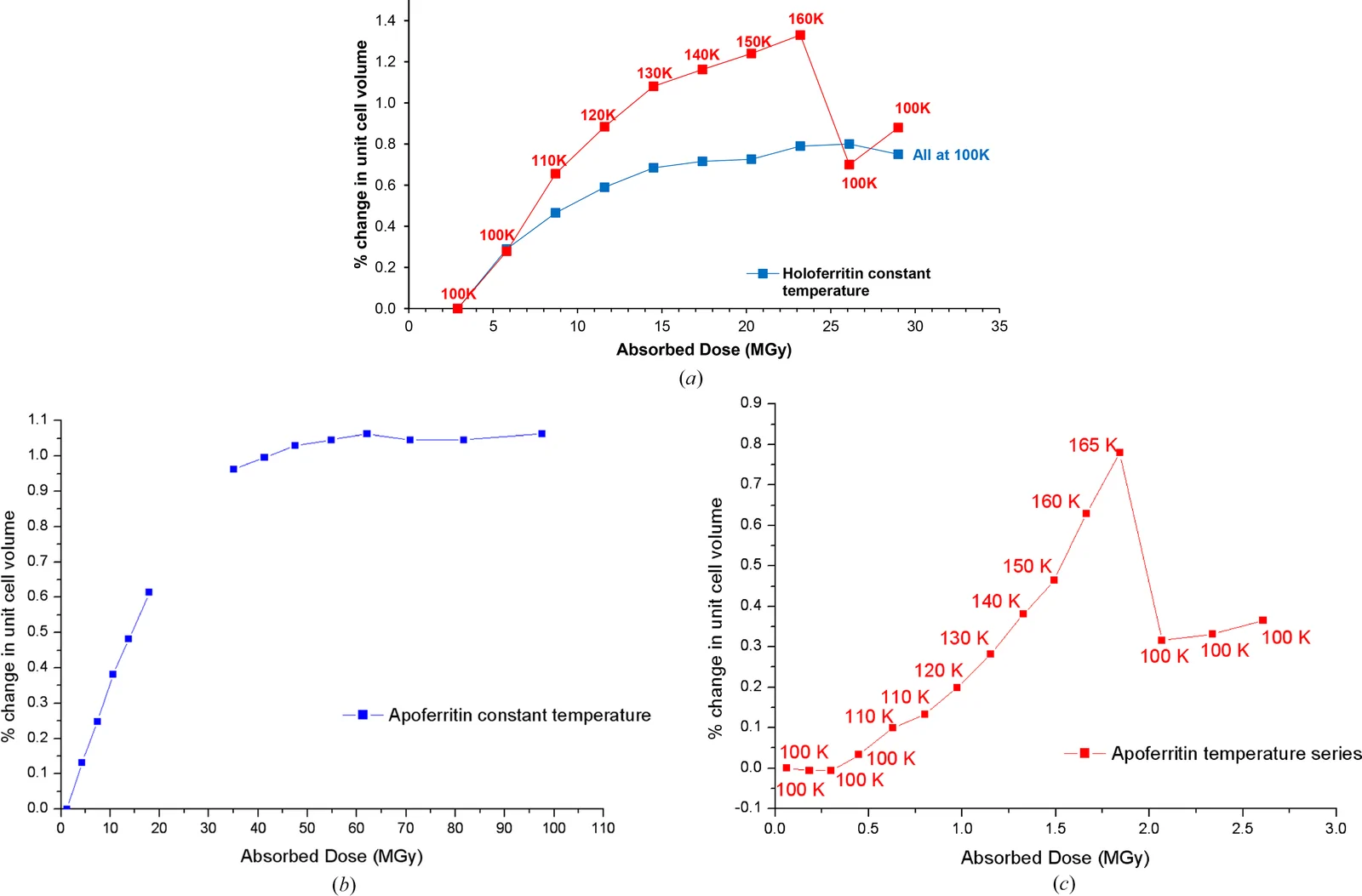
Changes in the unit-cell volume of ferritin crystals with temperature and absorbed X-ray dose. (*a*) Holoferritin constant series 1 (HCS_1) and holoferritin temperature series 1 (HTS_1) recorded on ID23-1 at ESRF. (*b*) Apoferritin constant series 1 (ACS_1) recorded on ID14-4 at ESRF. (DS-7 is missing because the images were not recorded.) The individual datasets were collected over 80 s at 20% transmission and the burns with unattenuated beam between each dataset for varying times: 4 s (burns after DS1 and DS2), 5 s (DS3 and DS4), 10 s (DS5, DS6 and DS7), 20 s (DS8 and DS9), 25 s (DS10 and DS11), 25 s (DS12), 35 s (DS13), 45 s (DS14) and 35 s (DS15). These increasing burn times account for the non-even spacing in dose of the dataset points above. (*c*) Apoferritin temperature series 1 (ATS_1) recorded on ID14-4 at ESRF. The purpose of the solid lines is to guide the eye; they are not fits. Linear extrapolation of the first four 100 K unit-cell volumes to 2.0 MGy falls below the later 100 K points, whereas if this extrapolation is restricted to the third and fourth 100 K values, it appears to approach the later values much more closely. The purpose of the solid lines is to guide the eye; they are not fits.

**Figure 3:**
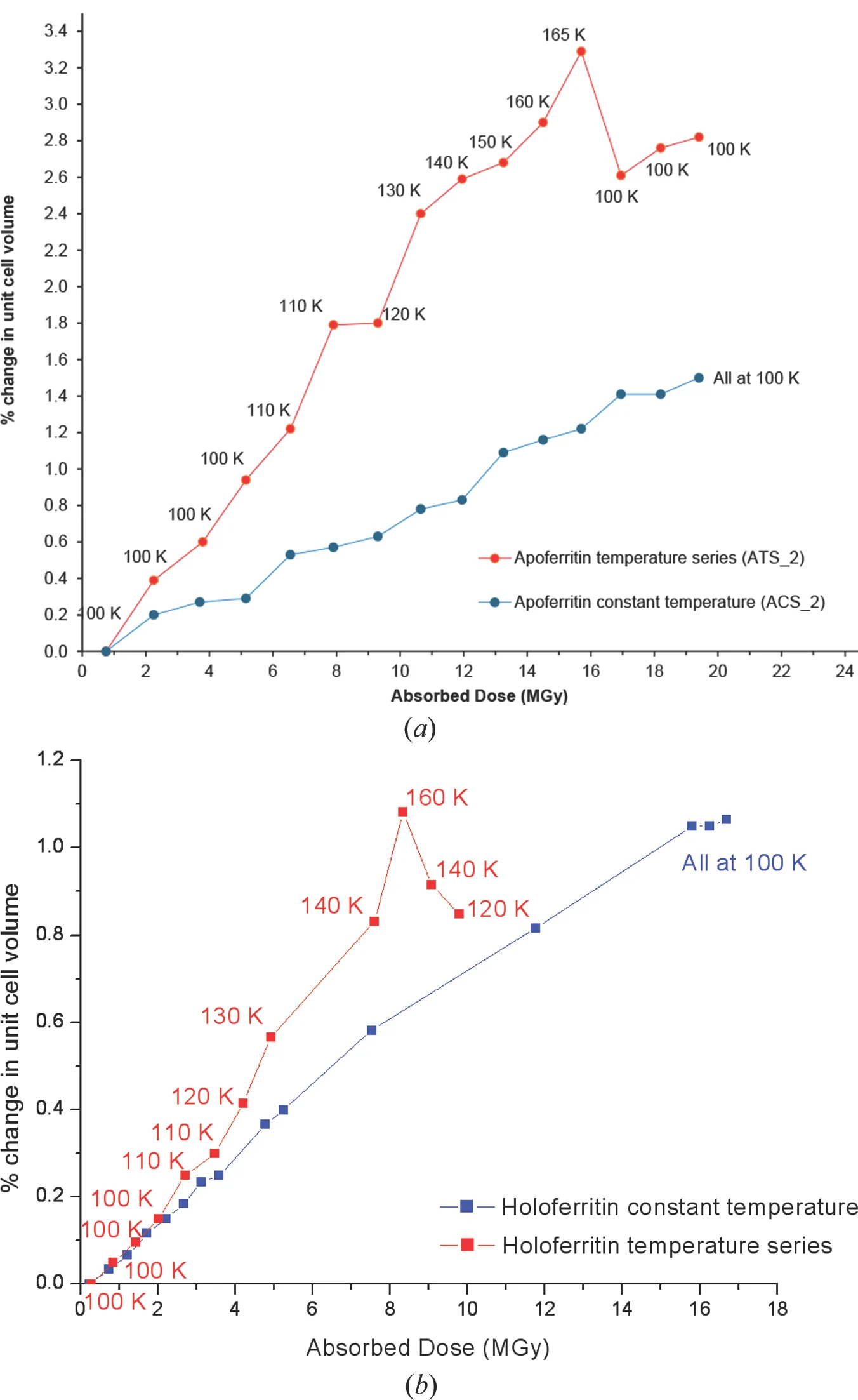
Changes in the unit-cell volume of more ferritin crystals with temperature and absorbed dose. (*a*) ACS_2 and ATS_2; (*b*) HCS_2 and HTS_2 all collected on ID14-4, ESRF. The purpose of the solid lines is to guide the eye; they are not fits.

The changes in unit-cell volume of apoferritin and holoferritin with dose under either a constant temperature series ‘CS’ (100 K) or under a varying temperature series ‘TS’ are shown in Figs. 2 ▸, 3 ▸ and 4 ▸. The corresponding data statistics are displayed in Supplementary Tables S1–S9, along with the refinement statistics for the structures deposited in the Protein Data Bank (Supplementary Tables S1*b*, S2*b* and S4*b*). In all cases, the expansion of unit-cell volume was greater in the TS than in the CS for an equivalent absorbed dose. The unit-cell variations of HCS_1 and HTS_1 are overlaid in Fig. 2 ▸(*a*), showing the reversible nature of the unit cell with temperature and the permanent change with dose. When the temperature was changed from 160 to 100 K in HTS_1 the unit-cell volume dropped very slightly below the corresponding HCS_1 value for the penultimate dataset, but subsequently rose above it for the last 100 K dataset. This undershoot on re-cooling (from 180 to 100 K) has been observed in other work (see Fig. 3*b* of Weik, Kryger *et al.*, 2001 ▸) and is explained by the densification of the solvent upon annealing owing to enthalpy relaxation: a process commonly observed in amorphous solids. The same effect was not observed for the ATS_2 data (Fig. 3 ▸*a*), since on cooling the crystal from 165 to 100 K the unit-cell volume did not decrease to the value for the ACS_2 cell at the equivalent dose (down to 2.6% of the starting unit-cell volume for ATS_2 compared with 1.4% for ACS_2). However, linear extrapolation of the first four ATS_2 100 K datapoints up to the last three collected at 100 K shows that the unit cell returned to approximately where it would have been if the crystal had remained at 100 K and not been subject to temperature ramping. It is notable that during the second, third and fourth datasets collected at 100 K the ATS_2 crystal unit cell expanded much more than during the first four datasets for ACS_2, reinforcing earlier observations on the variability of unit-cell increase for crystals of the same protein under the same conditions (Murray & Garman, 2002 ▸; Ravelli *et al.*, 2002 ▸).

ACS_1 (Fig. 2 ▸*b*) and ATS_1 (Fig. 2 ▸*c*) have not been overlaid because of the vastly different dose ranges encompassed. This dose mismatch was because the data for ACS_1 had initially been collected to establish a radiation dose limit at 100 K (Owen *et al.*, 2006 ▸) and had included a series of ‘burns’ (unattenuated beam of ∼1.3 ×10^12^ photons s^−1^; see the legend to Fig. 2 ▸*b*) between datasets collected with 25% transmission, whereas the ATS_1 data collection did not include full-beam burns between datasets. The ACS_1 crystal showed a linear increase in unit-cell volume of around 1% at a dose of ∼35 MGy, after which it levelled off with very little further expansion up to the maximum accumulated dose, in excess of 100 MGy. The ATS_1 crystal displayed an irreversible increase in unit-cell volume (0.35%) with dose but a reversible increase with temperature (0.8% at 165 K shrinking back to 0.3% at 100 K); at 165 K the 0.8% increase in unit-cell volume is assigned to a 0.5% irreversible expansion due to accumulated dose and a 0.3% reversible expansion due to temperature. Note that if the response with dose of the first three measurements at 100 K are linearly fitted and extrapolated up to the three further 100 K ATS_1 measurements above 2 MGy, the line comes near, although below, the latter results. This again indicates that the unit-cell volume increase with temperature is reversible.

The ACS_2, ATS_2, HCS_2 and HTS_2 data were all measured over approximately the same dose range; therefore, comparisons between these crystals were more straightforward (Fig. 3 ▸).

Comparison of the HCS_2 and HTS_2 plots in Fig. 3 ▸(*b*) shows similar behaviour to that of HCS_1 and HTS_1, except that the HTS_2 crystal was not returned to 100 K, only 120 K. It can be seen that extrapolating the 160–120 K plot further with dose would bring it very near the trend for the HCS_2 crystal. In addition, extrapolating the first three 100 K data points of HTS_2 gives a dose value at 10 MGy close to that for HCS_2. However, all these extrapolations of 100 K measurements are quite long-range and it is important to note that we would not expect them to coincide. Murray & Garman (2002 ▸) showed that for the three systems used in the current study (apoferritin, holoferritin and N9 neuraminidase), crystals of the same protein exhibited very different rates of unit-cell expansion, even when one large crystal was broken into smaller pieces and irradiated. This signalled that unit-cell expansion was not a robust radiation-damage metric to use as an indicator that data collection should stop once a certain percentage expansion had been reached.

**Figure 4:**
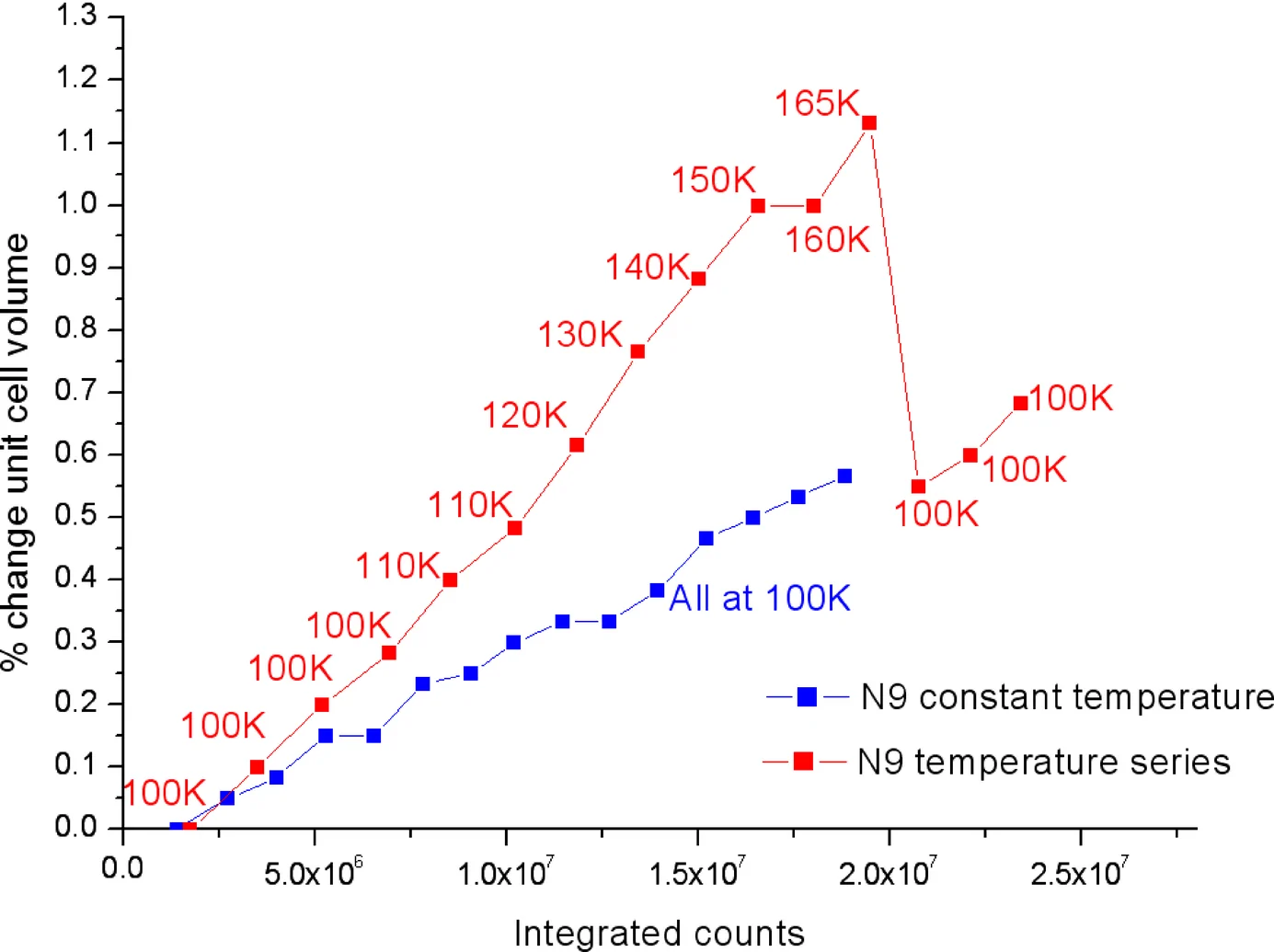
Changes in the unit-cell volume of N9 neuraminidase crystals with temperature and dose. Constant (N9CS) and variable temperature (N9TS) series recorded on ID14-4 at ESRF. The purpose of the solid lines is to guide the eye; they are not fits. Note that extrapolation of the first four 100 K data points on N9TS gives a slightly higher unit-cell volume than the values obtained when reducing from 165 K back to 100 K, again possibly due to solvent densification.

**Figure 5:**
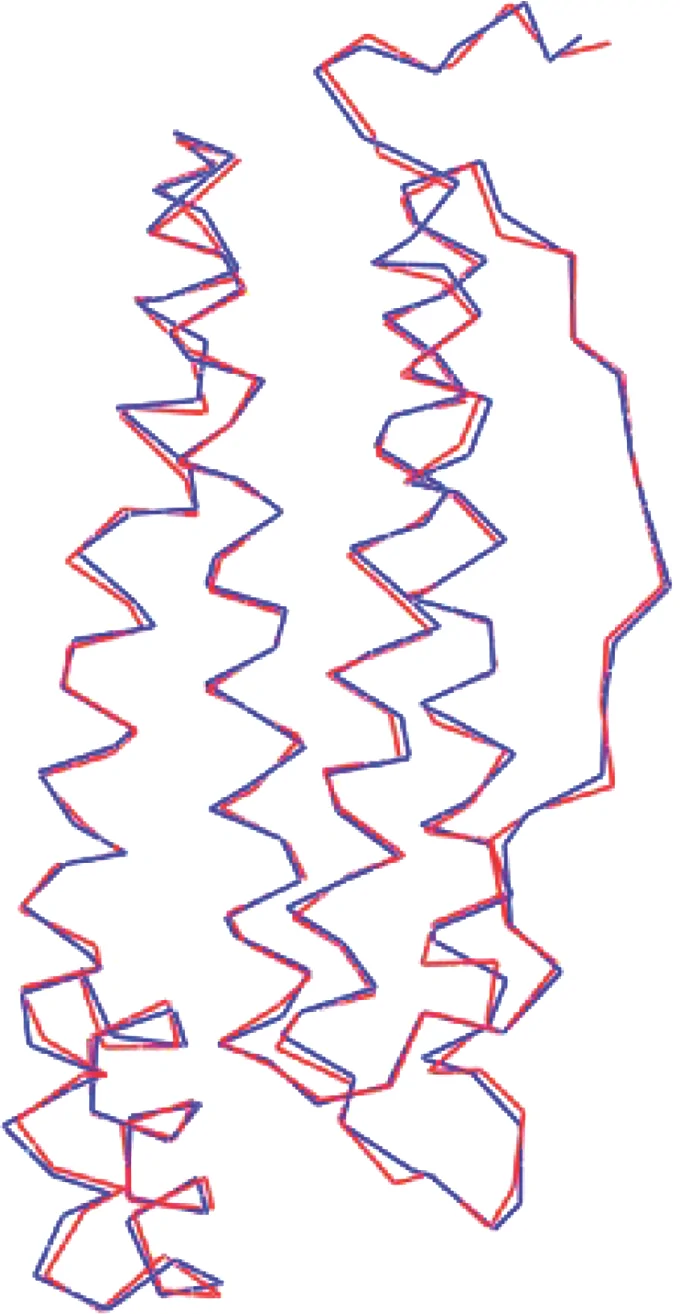
Effect on a holoferritin monomer of a temperature ramp and return. C^α^ traces of dataset 1 (100 K) shown in blue and of dataset 15 shown in red for ACS_1 (100 K). The movements of the individual monomers are visible but are small in magnitude.

The unit-cell volume of the *I*432 cubic system of N9 neuraminidase was plotted against integrated counts rather than absorbed dose (Fig. 5 ▸) since there was an issue with beam centering on ID14-4 and the crystals were only being hit by approximately one third of the beam. This meant that the crystal lifetimes were much longer than expected. However, the data from both crystals were collected under the same conditions so comparing the changes in unit-cell volume between crystals remains valid. It can be seen (Fig. 4 ▸) that the N9TS_1 crystal showed the same behaviour as that of holoferritin: following the temperature ramp from 100 to 165 K and back down in one step to 100 K, the unit-cell volume contracted and reached a value close to and a little below that of the N9CS_1 crystal.

The quality of the data statistics was degraded in all crystals as the absorbed dose increased (Supplementary Tables S1–S8). Both *I*/σ(*I*) and MnImean (the summed intensity of all reflections on a diffraction image) values decreased as data collection progressed, as observed by the fading of the diffraction spots. As already mentioned, the dose ranges experienced by the ACS_1 and ATS_1 crystals are very different. The larger absorbed dose (∼100 MGy) of the final dataset of ACS_1 resulted in lower *I*/σ(*I*) values, higher *R*_merge_ and Wilson *B* values, and larger relative *B* values between datasets, all indicating that this crystal suffered greater radiation damage than the ATS_1 crystal, which only absorbed a dose of 2.6 MGy.

The data statistics for HTS_1 (Supplementary Table S2*a*) are of poorer quality than for HCS_1 (Supplementary Table S1*a*), possibly due to the heating effect of the iron core at the elevated temperature of the HTS_1 crystal (Ravelli *et al.*, 2002 ▸).

### 3.2. Structural consequence of the changes in unit-cell volume

It is very clear from Figs. 2 ▸, 3 ▸ and 4 ▸ that the crystal unit-cell volumes changed during temperature changes and X-ray exposure. The magnitude of the changes ranged from −0.35% (shrinkage of HTS_2 on cooling from 160 to 120 K) to +2.4% (expansion of ATS_2 on heating from 100 to 165 K). What is not clear from these plots is how the individual monomers or the 24-mer balls were affected during this expansion. The distance between particular C^α^ atoms of a monomer (Table 2 ▸) and the diameter of the 24-mer balls (Table 3 ▸) was monitored to understand how the protein molecules are affected by dose and temperature. It is apparent that there is an expansion of individual monomers as well as an increase in the size of the 24-mer balls. A similar observation was made by Ravelli & McSweeney (2000 ▸), where a comparison of models refined for successive datasets showed that the molecules were slightly rotated (for example, a total rotation of 0.4° over 17 datasets for hen egg-white lysozyme crystals irradiated with an un­attenuated beam on ID14-4 at ESRF) and translated within the unit cell compared with the first model in each series.

Fig. 5 ▸ shows a C^α^ trace of the first (blue) and last (red) datasets of the structures derived from ACS_1. The slight displacement of atoms in the final data set may lead to a reduction in the signal strength of the difference maps. For ferritin with a unit-cell length of 183 Å, a 1% change in unit-cell volume as observed here in some of the crystals corresponds to a movement of 0.6 Å in atom position. When examining the distance between equivalent atom positions in dataset 1 and dataset 7 of HTS_1 (unit-cell volume change of ∼1.0%) there was a 0.5 Å average displacement of atoms, in agreement with the above prediction. One possible explanation of the 24-mer and unit-cell expansion is that the gas produced in irradiated crystals is trapped both inside the ferritin balls, causing the ball diameter to increase, and within the solvent between the balls, leading to unit-cell expansion.

### 3.3. Crystal morphology

**Figure 6:**
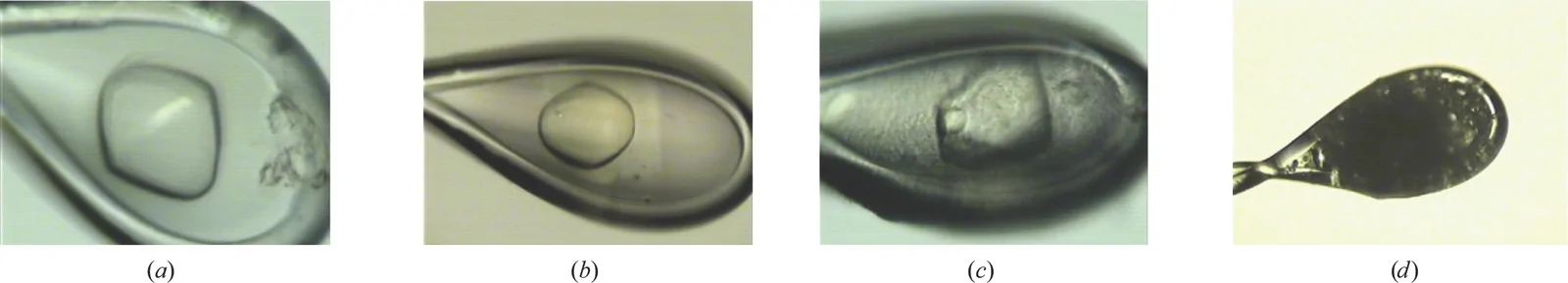
The degradation in crystal morphology with dose. (*a*) Apoferritin crystal before irradiation (crystal edges of ∼100 µm). (*b*) Irradiation caused the liquid in the loop to be bleached along the beam path. (*c*) The edges of the crystal degrade upon further exposure. (*d*) When the crystal is warmed up to 293 K the crystal explodes and bubbles of gas are released.

**Figure 7:**
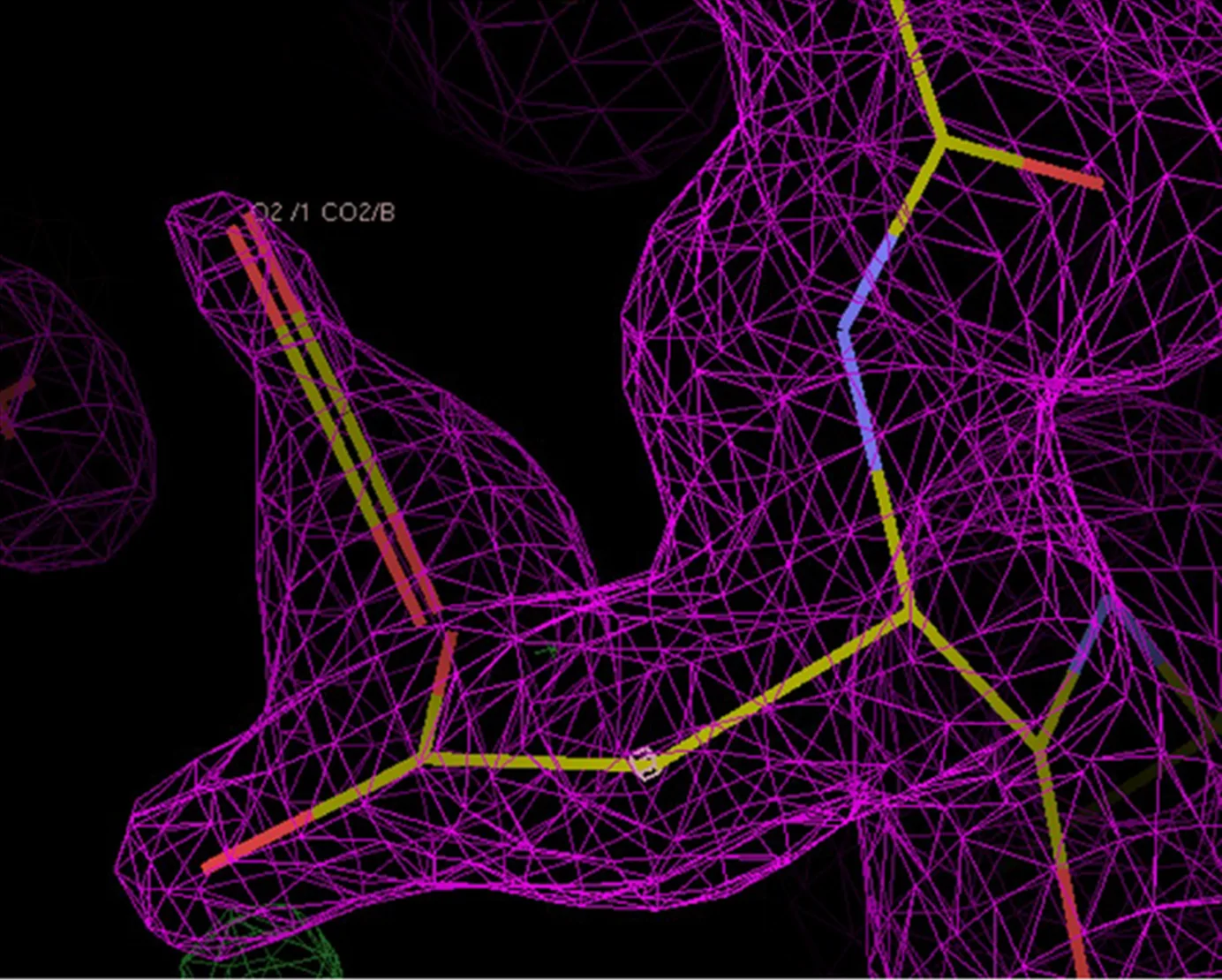
2*F*_o_ − *F*_c_ electron-density map consistent with a bound CO_2_ molecule (r.m.s.d. 0.3) next to Asp128 in subtype N9 neuraminidase (from noddy tern; PDB entry 6hfc, 1.29 Å resolution). The CO_2_ molecule was refined with 0.5 occupancy, as was the neighbouring partially decarboxylated Asp128 residue (*R*_free_ = 0.164, *R*_work_ = 0.141).

The morphology of all of the ferritin crystals altered considerably during X-ray exposure (Fig. 6 ▸). The crystal surface undergoes a characteristic degradation process in which the amorphous liquid surrounding the crystal, cooled to 100 K, is often bleached by the X-ray beam (Fig. 6 ▸*b*). Subsequently, the crystal surface becomes rough and its edges start to appear irregular (Fig. 6 ▸*c*). The final image (Fig. 6 ▸*d*) was taken after the crystal was removed from the cold gaseous nitrogen stream and then replaced into it after a few seconds of warming to RT (by which point the crystal had exploded and released bubbles of gas). The identity of this gas is very likely to be a mixture of molecular hydrogen with a trace of carbon dioxide from the decarboxylation of Asp and Glu residues. In fact, trapped CO_2_ molecules have been modelled in several 100 K crystal structures deposited in the PDB, although they are usually of low occupancy and may not be built into the models. For instance, two CO_2_ molecules were refined in a model of acetyl­cholinesterase by Colletier *et al.* (2008 ▸), as shown in Fig. S3 of their publication, but were not included in the final deposited structure (PDB entry 2vjb; Colletier *et al.*, 2008 ▸). Electron density consistent with a bound CO_2_ molecule has been observed in the 1.29 Å resolution electron-density map of N9 neuraminidase (from noddy tern, PDB entry 6hfc; M. T. Salinger, J. R. Hobbs, J. W. Murray, W. G. Laver, P. Kuhn & E. F. Garman, unpublished work) and refined with 0.5 occupancy for the C and two O atoms involved and 0.5 for the neighbouring partially decarboxylated Asp128 residue (*R*_free_ = 0.164, *R*_work_ = 0.141; Fig. 7 ▸).

### 3.4. The relationship between temperature, unit-cell volume and specific damage

A comparison was made between the structures derived from DS12 (PDB entry 31yy) and DS13 (PDB entry 31za) of ATS_1 to investigate how the magnitude of the peaks in the electron-density difference maps changed when the crystal temperature was decreased from 165 to 100 K. Although upon cooling the unit-cell volume contracted compared with its value in DS12 (0.8% to 0.35%; Fig. 2 ▸*c*), the peaks in the structure-factor difference maps (*F*_o1_ − *F*_o12_ versus *F*_o1_ − *F*_o13_, phases from the refined model obtained using DS1 as detailed above) did not become any smaller but in fact increased in magnitude between DS12 and DS13. This means that although there was a temperature change which allowed the unit cell to relax and the data statistics to marginally improve (for example a reduction in the Wilson *B* factor from 11.61 Å^2^ in DS12 to 9.62 Å^2^ for DS13), the specific damage continued to increase with the absorbed dose.

**Figure 8:**
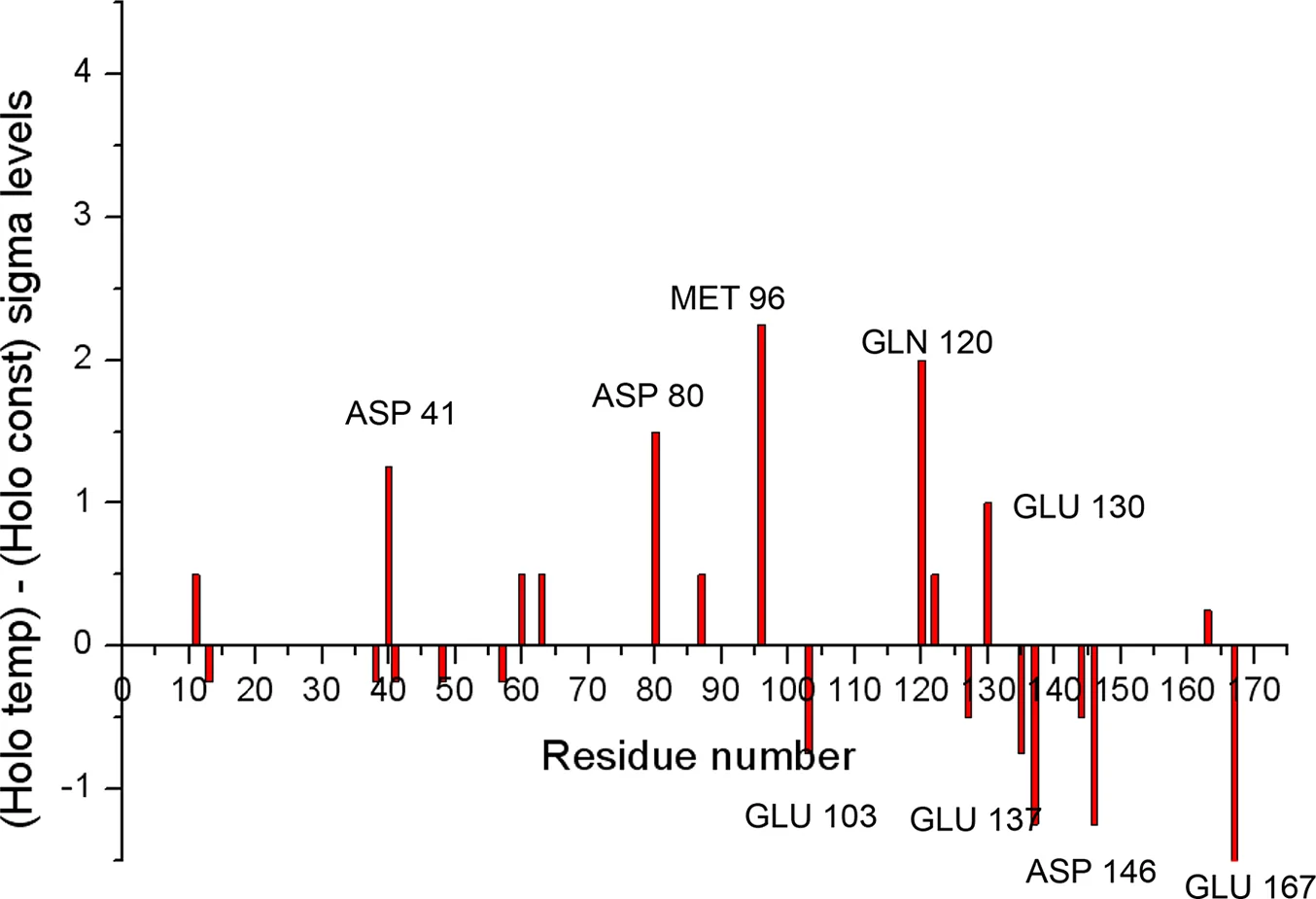
Plot showing the difference in the sigma levels of the electron-density difference maps for the HTS_2 dataset 9 (D9, 140 K) and HCS_2 dataset 11 (DS11, 100 K) against residue number. There are positive peaks where damage is greater for models from HTS_2 and negative peaks where damage was greater for HCS_2. These differences are taken from structure-factor difference maps produced after the same absorbed dose (7.5 MGy). The largest peaks in the difference map were predominantly Glu and Asp residues, apart from Gln120. Damage to Gln residues in data collected at 160 K was also noted by Juers & Weik (2011 ▸). Note that there are no disulfide bonds in ferritin.

A comparison was also made between structures derived from HTS_2 at 140 K (DS9) and those from HCS_2 at 100 K (DS11) at the same absorbed dose to try and disentangle the effect of temperature from that of dose (Fig. 8 ▸). There was greater damage seen for the HTS_2 model than for the HCS_2 model, presumably due to the heating effect of the iron core, which for holoferritin is predicted to be in excess of 100 K (Ravelli *et al.*, 2002 ▸). Under conditions of convective cooling, with the simplifying assumption of a lumped system where thermal gradients do not exist within the sample, the steady-state temperature rise was calculated to be 103 K on ID14-4 at 13.2 keV/0.939 Å for a holoferritin crystal of (350 µm)^3^, a 5 s exposure and a flux of 1 × 10^13^ photons s^−1^. This large rise in temperature is now thought to be an overestimate, and improved modelling using advanced computational fluid dynamics has predicted typical temperature rises of 5–10 K for a 200 µm diameter spherical biocrystal subjected to a 13 keV X-ray beam of 4 × 10^14^ photons s^−1^ mm^−2^ flux density (Mhaisekar *et al.*, 2005 ▸). However, no calculations were performed for holoferritin, which is an extreme case due to its iron core with one Fe atom for every two amino acids, giving it a very high absorption coefficient (apoferritin, 0.462 mm^−1^; holoferritin, 1.277 mm^−1^ at 12.7 keV).

**Figure 9:**
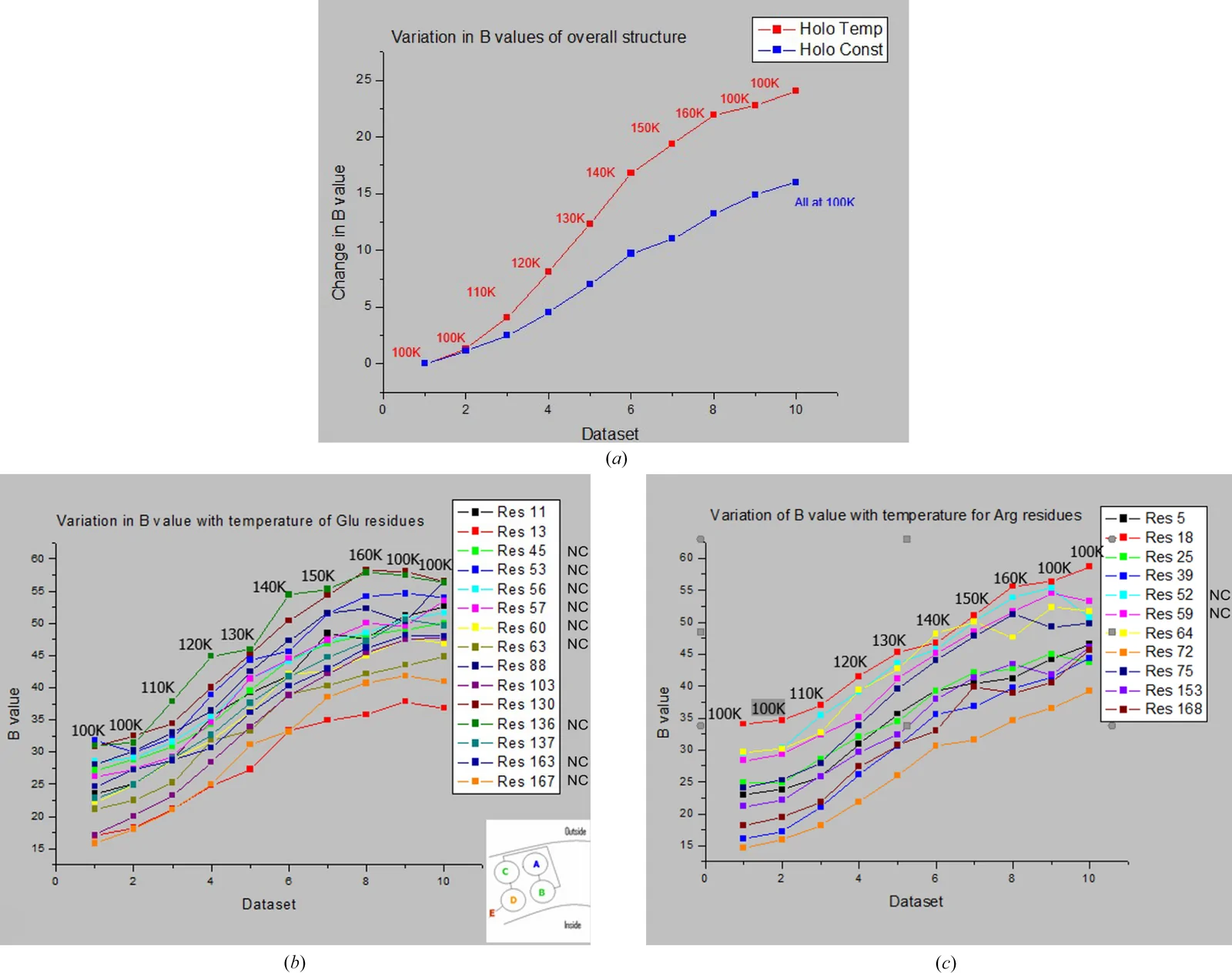
Atomic *B* factors of whole structures refined from the holoferritin constant series 1 (blue; HCS_1) and holoferritin temperature series 1 (red; HTS_1). *B* values are in units of Å^2^. (*b*) Variation in atomic *B* values of all Glu residues in holoferritin from HTS_1. In the inset, A, B, C and D are the four helices making up the holoferritin molecule. Residues close to the iron core are Glu45, Glu53, Glu56, Glu57, Glu60, Glu63, Glu136, Glu163 and Glu167. Those near the iron core are labelled ‘NC’. (*c*) Variation in atomic *B* factors of all Arg residues in holoferritin from HTS_1. Those near the iron core are labelled ‘NC’.

**Figure 10:**
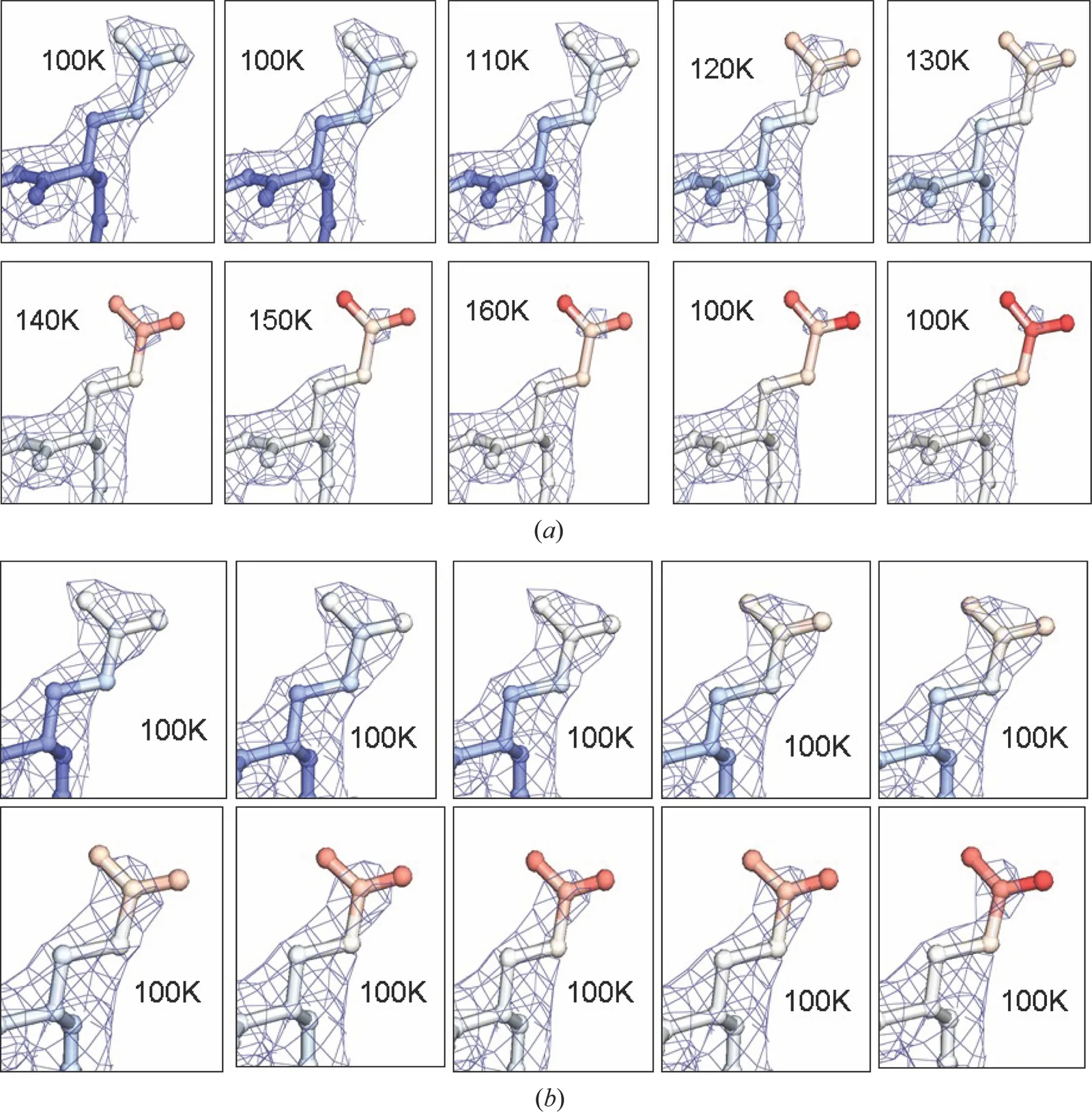
(*a*) Series of 2*F*_o_ − *F*_c_ electron-density maps (0.4 e Å^−3^) of Glu53 in the HTS_1 holoferritin temperature series of ten datasets as the temperature is raised from 100 K up to 160 K (DS8, ∼23.2 MGy) and then the change after being taken back down to 100 K (DS9, ∼26.1 MGy; DS10, ∼29.0 MGy). (*b*) Similar plots for HCS_1 all at 100 K. The atoms are coloured according to their atomic *B* factors from blue (10 Å^2^) to red (60 Å^2^). This figure was produced using *AESOP* (M. E. M. Noble, unpublished work).

It is well established that atomic *B* values increase as a function of dose, and in fact they can be deconvoluted from the local packing density to give ‘*B*_Damage_’ values for every atom (Gerstel *et al.*, 2015 ▸). These in turn can be used to estimate the overall level of damage that a 100 K structure has suffered by calculating the *B*_net_ metric from them (Shelley & Garman, 2022 ▸). The *B* values of the models refined from the ten datasets of HCS_1 and of HTS_1 were analysed and the results for the whole structure were plotted (Fig. 9 ▸*a*). It can be seen that for HCS_1 the *B*-value increase is approximately linear with dose, whereas each 10 K temperature rise induces larger increases than at 100 K, with these increases becoming smaller for the last two data sets collected at 100 K. For the more radiation-sensitive Glu residues the behaviour is slightly different as the temperature varies (see Fig. 9 ▸*b*). The *B* values rise as the temperature changes, especially for those residues closer to the inner layer of the ferritin ball that lie near to the highly absorbing atoms of iron in the core, such as Glu130 and Glu136. The *B* values of some Glu residues (Glu13, Glu53, Glu103, Glu136 and Glu167) respond to the decrease from 160 to 100 K by reducing, showing that some of the *B*-value rise due to temperature is reversible. For Arg residues (Fig. 9 ▸*c*), which are expected to be far less radiation-sensitive than Glu residues, the *B*-value increases are much more linear and do not respond to the decrease in temperature for the last two datasets, except for Arg52 and Arg59, which lie near the iron core. This implies that the iron core is affecting the behaviour of residues that are in close proximity to it. Figs. 10 ▸(*a*) and 10 ▸(*b*) show the electron density and atomic *B* factors of Glu63 in holoferritin for each of the ten datasets of HTS_1 and HCS_1. This residue was chosen as it is on the inside of the 24-mer near the iron core. It can be seen that the *B* factors do not decrease when the temperature is reduced from 160 to 100 K, the specific damage is not ‘repaired’ and the decarboxylation is more severe for the HTS_1 data than for HCS_1 at the same stages of irradiation.

## 4. Discussion

The results in this work highlight the relationship between dose, temperature, unit-cell volume and specific damage. There is an irreversible expansion of the unit-cell volume with dose and a reversible expansion with temperature increase (up to below 160 K). In all cases, the unit-cell volume was larger for the same absorbed dose when the temperature of the experiment was higher. The pattern of behaviour of the unit-cell volume was reproducibly observed in crystals of apo­ferritin, holoferritin and N9 influenza neuraminidase. We draw these conclusions despite the fact that for some crystals reduction from 160 or 165 K and back to 100 K did not result in a return of the unit-cell volume to exactly the same value as for the crystals irradiated at a constant 100 K. As mentioned above, it is well known that the unit-cell volumes of different crystals of the same protein expand at different rates (Murray & Garman, 2002 ▸), most likely related to their degree of internal order, so it would not be expected that there would be absolute agreement.

Bubbles of gas were visible upon warming the irradiated crystals from 100 K to RT, and the internal pressure following this cryo-trapped gas production seems to be the most likely cause of the unit-cell expansion with dose. In conjunction with this expansion of the unit-cell volume, there was degradation in the crystal morphology, coupled with an apparent translation of individual ferritin monomers within the unit cell and an expansion of the 24-mer balls leading to non-isomorphism between datasets. An electrostatic explanation is unlikely, as it has been estimated this could not provide enough force to explain the observed expansion (Garman and Ravelli, private communication).

There was no correlation between the temperature effects on unit-cell volume and the observed specific damage. When the temperature between two sequential datasets was decreased, the unit-cell volume decreased; however, the specific damage continued to increase with absorbed dose. A higher temperature dataset in holoferritin (140 K; Fig. 8 ▸) produced greater damage than a dataset at the equivalent absorbed dose in a crystal of holoferritin at 100 K, presumably due to the higher mobility of radical species above 100 K (Weik, Ravelli *et al.*, 2001 ▸) and sample-heating effects of the X-ray beam on the iron core (Ravelli *et al.*, 2002 ▸).

Up to 30 MGy, the magnitude of the difference map peaks continued to increase with dose (data not shown), as did the unit-cell volume. Above this dose, the unit-cell volume levelled off and the specific damage maps became much noisier. At this point the crystal had suffered significant radiation damage and scaling the reflections from datasets with such poor resolution became more problematic. The relationship between damage and dose appeared linear only up to the previously determined experimental limit (30 MGy; Owen *et al.*, 2006 ▸).

The careful characterization of radiation damage is essential in structural biology, as confident conclusions can then be drawn and the correct biological interpretation of X-raydiffraction-derived structure models. In particular, radiation-induced artefacts can be recognized and identified rather than being misassigned as biologically significant.

Structures derived from the first seven datasets of HCS_1, the first six of HTS_1 and all 15 from ATS_1 have been deposited in the PDB. The codes are listed below and are shown in the relevant Supplementary Tables (Supplementary Tables S1*b*, S2*b* and S4*b*, respectively). HCS_1: 32ch, 32ci, 32cj, 32cl, 32cw, 32dd, 32cm. HTS_1: 32co, 32cp, 32cq, 32dv, 32ca, 32et. ATS_1: 31xt, 31yb, 31ya, 31yo, 31yp, 32hl, 31yr, 31yu, 31yv, 31yw, 31yx, 31yy, 31za, 31zc, 31zd.

## Supplementary Material

PDB reference: HCS_1, 32ch

PDB reference: 32ci

PDB reference: 32cj

PDB reference: 32cl

PDB reference: 32cw

PDB reference: 32dd

PDB reference: 32cm

PDB reference: HTS_1, 32co

PDB reference: 32cp

PDB reference: 32cq

PDB reference: 32dv

PDB reference: 32ea

PDB reference: 32et

PDB reference: ATS_1, 31xt

PDB reference: 31yb

PDB reference: 31ya

PDB reference: 31yo

PDB reference: 31yp

PDB reference: 32hl

PDB reference: 31yr

PDB reference: 31yu

PDB reference: 31yv

PDB reference: 31yw

PDB reference: 31yx

PDB reference: 31yy

PDB reference: 31za

PDB reference: 31zc

PDB reference: 31zd

Supplementary Tables. DOI: 10.1107/S2059798326008612/cb5159sup1.pdf

## Figures and Tables

**Table 1:** Experimental parameters for all of the data series collected at the ESRF and reported here The detector on ID23-1 was a MAR MOSAIC 225 mm CCD (the first two series) and that on ID14-4 (the other eight series) was an ADSC Q315, which is a 3 × 3 CCD.

Sample	Crystal-to-detector distance (mm)	Incident X-ray energy (keV)	Δφ (°)	No. of images	Exposure time (s)	No. of datasets
Holoferritin constant series 1 (HCS_1)	240	13.2	1.0	30	2.0	10
Holoferritin temperature series 1 (HTS_1)	240	13.2	1.0	30	2.0	10
Apoferritin constant series 1 (ACS_1)	150	12.7	0.5	80	1.0	14
Apoferritin temperature series 1 (ATS_1)	190	12.7	0.6	60	1.5 (DS1–DS3), 2.5 (DS4–DS15)	15
Holoferritin constant series 2 (HCS_2)	252	12.7	1.0	25	1.0	15
Holoferritin temperature series 2 (HTS_2)	287	12.7	1.0	30	1.0	12
Apoferritin constant series 2 (ACS_2)	287	12.7	1.0	25	2.0 (DS1), 1.5 (DS2–DS15)	15
Apoferritin temperature series 2 (ATS_2)	287	12.7	1.0	25	1.5	15
N9 constant series (N9CS_1)	288	12.7	0.5	90	1.0	15
N9 temperature series (N9TS_1)	288	12.7	0.5	90	1.0	15

**Table 2:** C^α^–C^α^ interchain distances for the holoferritin temperature series 1 (HTS_1) monomer during data collection

	Interchain distance, dataset 1 (Å)	Interchain distance, dataset 7 (Å)	Net change in interchain distance (Å)
C^α^59–C^α^25	8.81	8.98	+0.17
C^α^55–C^α^29	7.58	8.45	+0.87
C^α^49–C^α^34	6.57	6.78	+0.21
C^α^66–C^α^16	8.11	8.80	+0.69
C^α^114–C^α^128	9.37	9.68	+0.31
C^α^110–C^α^134	7.12	7.47	+0.35
C^α^103–C^α^139	10.79	10.05	−0.74
C^α^96–C^α^148	6.28	6.65	+0.37
C^α^90–C^α^155	13.65	13.23	−0.42
C^α^128–C^α^65	9.10	9.47	+0.37
C^α^136–C^α^57	10.08	10.25	+0.17
C^α^147–C^α^47	11.06	11.05	−0.01
C^α^113–C^α^15	6.86	7.03	+0.17
C^α^26–C^α^102	7.07	7.28	+0.21

**Table 3:** C^α^64–C^α^53 distances for different crystals showing the expansion of the 24-mer ferritin ball during data collection The C^α^64 and C^α^53 atoms are located on symmetry-related monomers on opposite sides of the ferritin ball.

Crystal	DS	C^α^64–C^α^53 distance (Å)	C^α^49–C^α^53 distance (Å)	C^α^56–C^α^136 distance (Å)
ACS_1	1	79.97	83.35	85.70
	3	80.03	83.40	85.79
	6	80.04	83.34	85.78
	11	80.11	82.99	85.25
	15	80.15	83.32	85.64
HCS_1	1	80.11	83.46	85.76
	3	80.33	83.66	86.00
	6	80.53	83.77	86.13
	8	80.45	83.72	86.15
	9	80.46	83.73	86.15
HTS_1	1	79.97	83.34	85.56
	7	80.39	83.42	84.85
