# Supplementary material for: Separating the effects of temperature and absorbed X-ray dose on unit-cell volume
Source: Acta Crystallogr D Struct Biol. 2026 Sep 15;82(Pt 10):1155–68. doi: 10.1107/S2059798326008612 (PMC13632753; doi:10.1107/S2059798326008612)
Supplement: Supplementary file 1 [file d-82-01155-sup1.pdf]

Acta Cryst D

STRUCTURAL
BIOLOGY

Volume 82 (2026)

Supporting information for article:

Separating the effects of temperature and absorbed X-ray dose on unit-cell volume

Robert J. Southworth-Davies, James W. Murray, Ian Carmichael, Enrique Rudiño-Piñera, Martin Weik and Elspeth F. Garman

Table S1a: Holoferritin constant temperature series 1 (HCS_1) all at 100 K. Parameters after scaling data from the HCS_1 crystal collected on beamline ID23, ESRF. Resolution in all cases was 30.00 – 2.10 Å overall (2.21 – 2.10 Å outer shell). The dose values for HTS_1 and HCS_1 (Table S2) were inferred from the change in Wilson *B*-factors, $B_{rel} = B_n - B_1$ (Kmetko *et al.*, 2006) during HCS_2 in the following manner. The HCS_2 B_{rel} values were plotted against the dose values obtained from the RADDOSE original code and a linear fit (R2=0.9931) allowed a 'coefficient of sensitivity', $SAD = \Delta B_{rel} / 8\pi^2 \Delta D$ of 0.007 Å/MGy to be extracted. Since crystals of the same protein have been found to have similar values of SAD at cryotemperatures (Kmetko *et al.,* 2006), this value was used to estimate the doses for HTS_1 and HCS_1, despite it being rather lower than that observed for instance for hen egg white lysozyme. It should be noted that the original RADDOSE code output the maximum dose in the 21-voxel array into which the crystal was partitioned for the calculation.

DS	Dose MGy Temp	Unique reflec-tions	Multiplicity (2.21-2.10 Å)	*I/σI* (2.21-2.10 Å)	Com-plete-ness % (2.21-2.10 Å)	*Rmeas0* (2.21-2.10 Å)	Unit cell: Å	Wilson *B*-value: Å^2	*Mn Imean*	Wilson $B_{rel} = B_n - B_0$ (Å^2)
1	2.9 100 K	15401	6.2 (4.8)	18.3 (4.0)	99.5 (97.7)	0.09 (0.37)	181.46	27.1	1	0
2	5.8 100 K	15491	6.1 (4.6)	15.5 (2.4)	99.5 (97.5)	0.10 (-)	181.63	30.0	0.78	2.9
3	8.7 100 K	15537	6.1 (4.5)	13.4 (1.6)	99.5 (97.4)	0.12 (-)	181.74	32.6	0.72	5.5
4	11.6 100 K	15571	6.0 (4.3)	11.5 (1.1)	99.4 (97.0)	0.16 (-)	181.81	32.1	0.65	5.0
5	14.5 100 K	15583	6.0 (4.4)	11.5 (1.0)	99.4 (97.3)	0.15 (-)	181.87	35.7	0.61	8.6
6	17.4 100 K	15577	5.9 (4.2)	11.2 (0.7)	99.3 (96.7)	0.16 (-)	181.89	36.6	0.58	9.5
7	20.3 100 K	15631	5.9 (4.2)	9.6 (0.5)	99.3 (96.8)	0.19 (-)	181.89	39.9	0.52	12.8
8	23.2 100 K	15565	6.0 (4.4)	10.2 (0.7)	99.4 (97.1)	0.18 (-)	181.93	37.3	0.51	10.2
9	26.1 100 K	15583	6.0 (4.3)	9.7 (0.5)	99.3 (96.8)	0.20 (-)	181.94	38.6	0.47	11.5
10	29.0 100 K	15642	5.9 (4.2)	8.6 (0.3)	99.2 (96.4)	0.23 (-)	181.91	44.0	0.43	16.9

Table S1b: Holoferritin constant temperature series 1 (HCS_1) all at 100 K refinement statistics for structures deposited in the PDB. Structures were refined using all reflections from 104.83 Å to the limit noted in column 4 below. The same R_{free} set (5% of reflections) was used throughout.

DS	Dose MGy	Unique reflec-tions used	High resolu-tion refine-ment limit: Å	R_{cryst} %	R_{free} %	RMSD bond lengths: Å	RMSD bond angles: degrees	Mean atomic *B*-value: $Å^2$	PDB code
1	2.9	14627	2.10	19.1	23.0	1.34	0.014	26.46	32CH
2	5.8	12868	2.20	19.1	23.7	1.56	0.017	27.96	32CI
3	8.7	11644	2.28	19.5	23.6	1.36	0.013	29.66	32CJ
4	11.6	9244	2.47	19.2	23.6	1.25	0.012	32.45	32CL
5	14.5	8725	2.52	18.9	22.7	1.32	0.013	33.96	32CW
6	17.4	8160	2.58	19.7	23.5	1.30	0.011	35.78	32DD
7	20.3	7252	2.69	19.3	25.2	1.38	0.014	38.25	32CM

Table S2a: Holoferritin variable temperature series 1 (HTS_1). Parameters after scaling data from the HTS_1 crystal collected on beamline ID23, ESRF. Resolution in all cases was 30.00 – 2.10 Å overall (2.21 – 2.10 Å outer shell). R values with a "-" symbol represent values above 50%.

DS	Dose MGy<br>Temp	Unique reflec-tions	Multiplicity (2.21-2.10 Å)	*I/σI* (2.21-2.10 Å)	Com-plete-ness % (2.21-2.10 Å)	*Rmeas0* (2.21-2.10)	Unit cell: Å	Wilson *B* - value (Å^2)	*Mn Imean*	Wilson *Brel* = B_n-B_0 (Å^2)
1	2.9<br>100 K	15346	5.6 (4.3)	13.8 (2.5)	99.7<br>(98.5)	0.11<br>(0.50)	181.09	25.4	1	0
2	5.8<br>100 K	15365	5.7 (4.3)	11.2 (1.4)	99.8<br>(98.9)	0.15 (-)	181.26	32.2	0.98	6.8
3	8.7<br>110 K	15301	5.5 (4.1)	7.7 (0.3)	99.7<br>(98.5)	0.27 (-)	181.48	45.9	0.73	20.5
4	11.6<br>120 K	15291	5.5 (4.0)	6.7 (0.1)	99.7<br>(98.5)	0.33 (-)	181.62	54.7	0.62	29.3
5	14.5<br>130 K	15205	5.1 (3.8)	5.3 (0.1)	99.4<br>(97.1)	0.41 (-)	181.74	56.8	0.41	31.4
6	17.4<br>140 K	15274	5.0 (3.7)	4.9 (0.1)	99.6<br>(98.0)	0.42 (-)	181.79	53.5	0.38	28.1
7	20.3<br>150 K	15219	5.0 (3.7)	3.8 (0.1)	99.6<br>(98.1)	0.53 (-)	181.83	49.9	0.24	24.5
8	23.2<br>160 K	15461	5.0 (3.7)	7.6 (0.2)	99.2<br>(96.4)	0.25 (-)	181.88	45.2	0.48	18.8
9	26.1<br>100 K	15090	4.9 (3.5)	4.1 (0.1)	98.9<br>(94.7)	- (-)	181.51	51.7	0.28	26.3
10	29.0<br>100 K	15152	5.0 (3.6)	4.3 (0.1)	99.3<br>(96.5)	- (-)	181.62	26.7	0.25	1.3

Table S2b: Holoferritin variable temperature series 1 (HTS_1) refinement statistics for structures deposited in the PDB. Structures were refined using all reflections from 104.83 Å to the limit noted in column 4 below. The same R_{free} set (5% of reflections) was used throughout.

DS	Dose MGy	Unique reflec-tions used	High resolu-tion refine-ment limit: Å	R_{cryst} %	R_{free} %	RMSD bond lengths: Å	RMSD bond angles: degrees	Mean atomic *B*-value: $Å^2$	PDB code
1	2.9	12607	2.21	19.1	22.8	1.14	0.010	22.21	32CO
2	5.8	10860	2.33	17.9	21.8	1.50	0.016	23.90	32CP
3	8.7	8900	2.50	18.5	21.7	1.27	0.012	26.50	32CQ
4	11.6	9244	2.64	18.3	22.8	1.25	0.012	30.30	32DV
5	14.5	8725	2.79	17.7	22.2	1.49	0.016	33.75	32EA
6	17.4	8160	2.90	18.3	24.7	1.42	0.013	35.86	32ET

Table S3: Apoferritin constant temperature series 1 (ACS_1) all at 100 K. Parameters after scaling data from the ACS_1 crystal collected on beamline ID14-4, ESRF. Resolution in all cases was 30.00 – 2.10 Å overall (2.21 – 2.10 Å outer shell). %. n/a represents data that were not recorded.

DS	Dose MGy Temp	Unique reflec-tions	Multiplicity (2.21-2.10 Å)	*I/σI* (2.21-2.10 Å)	Com-plete-ness % (2.21-2.10 Å)	*Rmeas0* (2.21-2.10 Å)	Unit cell Å	Wilson *B* - value ($Å^2$)	*Mn Imean*	Wilson $B_{rel} = B_n - B_0$ ($Å^2$)
1	1.2 100 K	15416	9.1 (9.3)	36.8 (19.3)	99.7 (100)	0.05 (0.10)	180.70	20.4	1	0
2	4.3 100 K	15425	9.1 (9.3)	36.9 (18.8)	99.8 (100)	0.05 (0.10)	180.78	20.9	0.86	0.5
3	7.4 100 K	15444	9.1 (9.3)	36.2 (17.8)	99.8 (100)	0.05 (0.11)	180.85	21.6	0.95	1.2
4	10.6 100 K	15448	9.1 (9.3)	35.8 (16.6)	99.8 (100)	0.05 (0.12)	180.93	22.7	0.89	2.3
5	13.8 100 K	15465	9.1 (9.3)	34.9 (15.5)	99.8 (100)	0.05 (0.14)	181.01	23.4	0.87	3.0
6	17.9 100 K	15458	9.1 (9.2)	31.8 (12.5)	99.9 (100)	0.06 (0.17)	181.07	25.3	0.81	4.9
7	27.4 100 K	n/a	n/a	n/a	n/a	n/a	n/a	n/a	n/a	n/a
8	35.0 100K	15427	9.0 (9.1)	16.8 (3.2)	99.9 (100)	0.11 (-)	181.29	36.6	0.60	16.1
9	41.3 100 K	15403	8.9 (9.0)	14.1 (1.8)	99.9 (100)	0.15 (-)	181.31	40.8	0.49	20.4
10	47.5 100 K	15419	8.9 (8.8)	14.1 (1.5)	99.9 (100)	0.15 (-)	181.33	42.5	0.52	22.1
11	54.8 100 K	15366	8.7 (8.7)	10.9 (0.6)	99.9 (100)	0.21 (-)	181.34	50.2	0.32	29.8
12	62.1 100 K	15376	8.5 (8.2)	11.9 (0.6)	100 (100)	0.19 (-)	181.35	51.6	0.32	31.2
13	70.8 100 K	15371	8.7 (8.3)	11.5 (0.4)	100 (100)	0.21 (-)	181.34	55.0	0.28	34.6
14	81.7 100 K	15372	8.6 (8.2)	11.3 (0.4)	100 (100)	0.22 (-)	181.35	54.0	0.27	33.6
15	97.6 100 K	15516	8.6 (8.2)	13.4 (1.0)	100 (100)	0.21 (-)	181.36	36.2	0.24	15.8

Table S4a: Apoferritin variable temperature series 1 (ATS_1). Parameters after scaling data from the ATS_1 crystal collected on beamline ID14-4, ESRF. Resolution in all cases was 30.00 – 2.10 Å overall (2.21 – 2.10 Å outer shell).

DS	Dose MGy Temp	Unique reflec-tions	Multiplicity (2.21-2.10 Å)	*I/σI* (2.21-2.10 Å)	Com-plete-ness % (2.21-2.10 Å)	*Rmeas0* (2.21-2.10 Å)	Unit cell Å	Wilson *B* value (Å^2)	*Mn Imean*	Wilson $B_{rel} = B_n - B_0$ (Å^2)
1	0.06 100 K	15435	7.7 (6.4)	22.0 (4.5)	100 (100)	0.09 (0.40)	181.22	22.8	1	0
2	0.18 100 K	15464	7.7 (6.6)	21.5 (4.3)	100 (100)	0.10 (0.43)	181.28	23.3	0.87	0.5
3	0.30 100 K	15446	7.7 (6.5)	21.9 (4.3)	100 (100)	0.10 (0.43)	181.24	23.5	0.90	0.7
4	0.45 100 K	15464	7.7 (6.5)	24.9 (5.9)	100 (100)	0.08 (0.32)	181.31	22.3	1.48	-0.5
5	0.63 110 K	15488	7.7 (6.6)	25.3 (5.8)	100 (100)	0.08 (0.33)	181.38	22.2	1.39	-0.6
6	0.80 110 K	15488	7.7 (6.6)	25.1 (5.7)	100 (100)	0.08 (0.33)	181.39	22.7	1.35	-0.1
7	0.97 120 K	15508	7.7 (6.5)	25.0 (5.7)	100 (100)	0.08 (0.33)	181.47	22.4	1.41	-0.4
8	1.15 130 K	15509	7.7 (6.5)	25.7 (5.8)	100 (100)	0.08 (0.33)	181.49	22.6	1.43	-0.2
9	1.33 140 K	15532	7.7 (6.6)	24.7 (5.4)	100 (100)	0.08 (0.36)	181.57	23.2	1.27	0.4
10	1.49 150 K	15540	7.7 (6.5)	23.6 (4.8)	100 (100)	0.08 (0.40)	181.62	23.3	1.19	0.5
11	1.66 160 K	15566	7.7 (6.5)	24.0 (4.8)	100 (100)	0.08 (0.41)	181.77	23.5	1.25	0.7
12	1.84 165 K	15604	7.7 (6.4)	23.1 (4.4)	100 (100)	0.08 (0.44)	181.91	24.1	1.22	1.3
13	2.07 100 K	15551	7.7 (6.5)	25.0 (5.2)	100 (100)	0.08 (0.37)	181.64	24.4	1.79	1.6
14	2.34 100 K	15556	7.7 (6.5)	24.7 (5.0)	100 (100)	0.08 (0.38)	181.69	24.5	1.63	1.7
15	2.61 100 K	15569	7.7 (6.5)	24.2 (4.8)	100 (100)	0.08 (0.39)	181.76	25.0	1.97	2.2

Table S4b: Apo variable temperature series 1 (ATS_1) refinement statistics for structures deposited in the PDB. Structures were refined using all reflections from 104.83 Å to the limit noted in column 4 below. The same R_{free} set (5% of reflections) was used throughout.

DS	Dose MGy	Unique reflec-tions used	High resolu-tion refine-ment limit: Å	R_{cryst} %	R_{free} %	RMSD bond lengths: Å	RMSD bond angles: degrees	Mean atomic *B*-value: $Å^2$	PDB code
1	0.06	13687	2.15	19.1	21.8	1.64	0.017	20.09	31XT
2	0.18	13711	2.15	19.3	21.8	1.64	0.018	20.43	31YB
3	0.30	13692	2.15	19.4	22.2	1.66	0.017	20.45	31YA
4	0.45	13678	2.15	19.4	22.5	1.66	0.018	19.94	31YO
5	0.63	13742	2.15	19.4	22.6	1.66	0.018	19.77	31YP
6	0.80	13736	2.15	19.3	21.8	1.64	0.017	20.33	32HL
7	0.97	13767	2.15	19.3	21.7	1.66	0.018	20.23	31YR
8	1.15	13741	2.15	19.1	22.3	1.66	0.018	21.04	31YU
9	1.33	13784	2.15	19.2	21.5	1.65	0.018	20.88	31YV
10	1.49	13762	2.15	19.3	21.9	1.65	0.018	21.23	31YW
11	1.66	13819	2.15	19.4	21.7	1.63	0.018	21.49	31YX
12	1.84	13845	2.15	19.7	22.5	1.69	0.018	22.79	31YY
13	2.07	13789	2.15	20.1	22.7	1.66	0.018	22.52	31ZA
14	2.34	13817	2.15	20.1	23.3	1.69	0.019	22.74	31ZC
15	2.61	13825	2.15	20.0	23.4	1.70	0.019	23.10	31ZD

Table S5: Apoferritin constant temperature series 2 (ACS_2) all at 100 K. Parameters after scaling data from the ACS_2 crystal collected on beamline ID14-4, ESRF Resolution in all cases was 30.00 – 2.30 Å overall (2.40 – 2.30 Å outer shell). R values with a "-" symbol represent values above 50%.

DS	Dose MGy Temp	Unique reflec-tions	Multiplic-ity (2.40-2.30 Å)	*I/σI* (2.40-2.30 Å)	Com-plete-ness % (2.40-2.30 Å)	*Rmeas0* (2.40-2.30 Å)	Unit cell Å	Wilson *B*-value (Å^2)	*Mn Imean*	Wilson $B_{rel} = B_n - B_0$ (Å^2)
1	1.0 100 K	11825	5.4 (5.4)	14.5 (4.8)	99.9 (100)	0.13 (0.31)	181.13	26.8	1	0
2	2.8 100 K	11841	5.4 (5.5)	14.2 (4.0)	99.9 (100)	0.13 (0.39)	181.25	29.2	0.75	2.4
3	4.3 100 K	11862	5.3 (5.4)	12.2 (3.0)	99.9 (100)	0.16 (0.48)	181.29	30.2	0.56	3.4
4	5.8 100 K	11882	5.3 (5.3)	13.0 (3.0)	99.9 (100)	0.14 (-)	181.31	30.7	1.03	3.9
5	7.3 100 K	11887	5.2 (5.0)	13.5 (2.8)	99.9 (100)	0.13 (-)	181.45	32.2	0.58	5.4
6	8.9 100 K	11888	5.3 (5.4)	11.8 (2.1)	99.9 (100)	0.16 (-)	181.48	35.5	0.58	7.7
7	10.4 100 K	11905	5.2 (5.1)	11.2 (1.7)	99.9 (100)	0.17 (-)	181.51	37.5	0.36	10.7
8	11.9 100 K	11911	5.2 (5.1)	10.9 (1.5)	99.9 (100)	0.18 (-)	181.60	40.0	0.65	13.2
9	13.4 100 K	11918	5.1 (4.9)	9.9 (1.1)	99.9 (100)	0.20 (-)	181.63	42.9	0.32	15.1
10	14.8 100 K	11943	5.1 (4.8)	10.0 (0.9)	99.9 (100)	0.20 (-)	181.79	45.2	0.34	18.6
11	16.3 100 K	11944	5.0 (4.8)	9.5 (0.7)	99.9 (100)	0.22 (-)	181.83	47.0	0.45	20.2
12	17.8 100 K	11991	5.0 (4.7)	9.5 (0.6)	99.9 (100)	0.22 (-)	181.87	48.3	0.37	21.5
13	19.3 10) K	11976	5.0 (4.7)	8.9 (0.5)	99.9 (100)	0.24 (-)	181.98	50.3	0.50	23.5
14	20.8 100 K	11993	5.0 (4.7)	8.3 (0.4)	99.9 (100)	0.26 (-)	181.98	52.3	0.25	25.5
15	22.2 100 K	11962	4.9 (4.6)	8.0 (0.4)	99.9 (99.9)	0.28 (-)	182.04	50.3	0.34	23.5

Table S6: Apoferritin variable temperature series 2 (ATS_2) Parameters after scaling data from the ATS_2 crystal collected on beamline ID14-4, ESRF. Resolution in all cases was 30.00 – 2.30 Å overall (2.40 – 2.30 Å outer shell). R values with a "-" symbol represent values above 50%.

DS	Dose MGy<br>Temp	Unique reflec-tions	Multiplicity (2.40-2.30 Å)	*I/σI* (2.40-2.30 Å)	Com-plete-ness % (2.40-2.30 Å)	*Rmeas0* (2.40-2.30 Å)	Unit Cell Å	Wilson *B*-value (Å^2)	*Mn Imean*	Wilson $B_{rel} = B_n - B_0$ (Å^2)
1	0.75<br>100 K	11799	5.5 (5.7)	15.9<br>(6.2)	99.9<br>(100)	0.10<br>(0.29)	180.92	23.6	1	0
2	2.25<br>100 K	11844	5.5 (5.7)	14.9<br>(5.5)	99.9<br>(100)	0.11<br>(0.32)	181.15	24.1	1.22	0.5
3	3.7<br>100 K	11867	5.5 (5.7)	14.2<br>(4.9)	99.9<br>(100)	0.11<br>(0.37)	181.28	26.3	0.64	2.7
4	5.15<br>100 K	11894	5.5 (5.7)	13.0<br>(4.2)	99.9<br>(100)	0.13<br>(0.42)	181.48	26.1	0.58	2.5
5	6.55<br>110 K	11925	5.5 (5.7)	13.1<br>(3.6)	99.9<br>(99.9)	0.13 (-)	181.65	28.0	0.63	4.4
6	7.9<br>110 K	11971	5.5 (5.7)	12.2<br>(3.1)	99.9<br>(100)	0.14 (-)	181.99	28.8	0.58	5.2
7	9.3<br>120 K	11985	5.5 (5.7)	11.8<br>(2.7)	99.9<br>(100)	0.15 (-)	182.00	31.1	0.56	7.5
8	10.65<br>130 K	12054	5.5 (5.7)	11.2<br>(2.3)	99.9<br>(99.9)	0.16 (-)	182.35	32.3	0.44	8.7
9	11.95<br>140 K	12132	5.5 (5.7)	10.8<br>(1.8)	99.9<br>(100)	0.18 (-)	182.46	34.7	0.42	11.1
10	13.25<br>150 K	12102	5.5 (5.7)	10.0<br>(1.2)	99.9<br>(100)	0.19 (-)	182.52	40.1	0.33	16.5
11	14.5<br>160 K	12125	5.5 (5.7)	9.0<br>(0.8)	99.9<br>(100)	0.23 (-)	182.65	42.7	0.28	19.1
12	15.7<br>165 K	12163	5.5 (5.7)	8.0<br>(0.6)	99.9<br>(100)	0.27 (-)	182.88	45.6	0.21	22.0
13	16.95<br>100 K	12085	5.5 (5.6)	7.4<br>(0.5)	99.9<br>(100)	0.30 (-)	182.48	44.9	0.20	21.3
14	18.2<br>100 K	12122	5.5 (5.6)	7.4<br>(0.5)	99.9<br>(100)	0.30 (-)	182.57	46.7	0.22	23.1
15	19.4<br>100 K	12121	5.5 (5.6)	7.3<br>(0.5)	99.9<br>(99.9)	0.32 (-)	182.61	45.6	0.21	22.0

Table S7: Holoferritin constant temperature series 2 (HCS_2) all at 100 K. Parameters after scaling data from the HCS_2 crystal collected on beamline ID14-4, ESRF. Resolution in all cases was 30.00 – 2.30 Å overall (2.40 – 2.30 Å outer shell).

DS	Dose MGy<br><br>Temp	Unique reflec-tions	Multiplic-ity (2.40-2.30 Å)	*I/σI* (2.40-2.30 Å)	Com-plete-ness % (2.40-2.30 Å)	*Rmeas0* (2.40-2.30 Å)	Unit cell Å	Wilson ***B***-value (Å^2)	*Mn Imean*	Wilson $B_{rel} = B_n - B_0$ (Å^2)
1	0.24<br>100 K	11727	5.6 (5.9)	15.9<br>(10.2)	99.9<br>(99.9)	0.11<br>(0.14)	180.63	18.0	1	0
2	0.73<br>100 K	11728	5.6 (5.8)	17.6<br>(11.3)	99.9<br>(99.9)	0.09<br>(0.12)	180.64	18.7	0.92	0.7
3	1.22<br>100 K	11734	5.6 (5.9)	18.5<br>(11.7)	99.9<br>(99.9)	0.09<br>(0.12)	180.66	18.7	1.02	0.7
4	1.72<br>100 K	11748	5.6 (5.8)	19.5<br>(12.2)	99.9<br>(99.9)	0.08<br>(0.12)	180.70	19.1	1.20	1.1
5	2.21<br>100 K	11747	5.6 (5.8)	18.3<br>(11.3)	99.9<br>(99.9)	0.09<br>(0.12)	180.71	18.9	1.91	0.9
6	2.66<br>100 K	11748	5.6 (5.8)	19.6<br>(11.9)	99.9<br>(99.9)	0.08<br>(0.12)	180.72	19.1	1.01	1.1
7	3.11<br>100 K	11748	5.6 (5.8)	19.1<br>(11.5)	99.9<br>(99.9)	0.09<br>(0.12)	180.73	19.5	1.08	1.5
8	3.58<br>100 K	11748	5.6 (5.8)	19.8<br>(11.7)	99.9<br>(99.9)	0.09<br>(0.12)	180.74	19.6	0.98	1.6
9	4.78<br>100 K	11754	5.6 (5.8)	18.9<br>(10.7)	99.9<br>(99.9)	0.09<br>(0.14)	180.77	20.4	0.96	2.4
10	5.24<br>100 K	11774	5.6 (5.8)	19.1<br>(10.7)	99.9<br>(99.9)	0.07<br>(0.14)	180.80	20.4	1.03	2.4
11	7.5<br>100 K	11780	5.6 (5.8)	17.6<br>(9.0)	99.9<br>(99.9)	0.08<br>(0.17)	180.86	22.2	1.03	4.2
12	11.76<br>100 K	11789	5.5 (5.8)	15.1<br>(6.5)	99.9<br>(99.9)	0.09<br>(0.26)	180.89	23.6	0.79	5.6
13	15.8<br>100 K	11799	5.4 (5.6)	12.6<br>(4.3)	99.9<br>(99.9)	0.12<br>(0.42)	180.96	26.2	0.72	8.2
14	16.25	11800	5.4 (5.6)	12.3<br>(4.1)	99.9<br>(99.9)	0.12<br>(0.45)	180.96	26.8	0.70	8.8
15	16.69	11799	5.4 (5.7)	12.4<br>(4.0)	99.9<br>(99.9)	0.12<br>(0.45)	180.98	27.0	0.72	9.0

Table S8: Holoferritin variable temperature series 2 (HTS_2) Holo temp series 2. Parameters after scaling data from the HTS_2 crystal collected on beamline ID14-4, ESRF. Resolution in all cases was 30.00 – 2.30 Å overall (2.40 – 2.30 Å outer shell). R values with a "-" symbol represent values above 50%. n/a represents data that were not recorded.

DS	Dose MGy<br><br>Temp	Unique reflec-tions	Multiplic-ity (2.40-2.30 Å)	*I/σI* (2.40-2.30 Å)	Com-plete-ness % (2.40-2.30 Å)	*Rmeas0* (2.40-2.30 Å)	Unit cell Å	Wilson *B* - value (Å^2)	*Mn Imean*	Wilson $B_{rel} = B_n - B_0$ (Å^2)
1	0.29<br>100 K	11736	6.7 (7.0)	24.6<br>(15.5)	99.9<br>(100)	0.06<br>(0.10)	180.69	21.2	1	0
2	0.85<br>100 K	11736	6.7 (7.0)	22.3<br>(14.0)	99.9<br>(100)	0.07<br>(0.11)	180.72	21.4	1.06	0.2
3	1.42<br>100 K	11736	6.7 (7.0)	25.1<br>(15.7)	99.9<br>(100)	0.06<br>(0.10)	180.75	21.5	1.10	0.3
4	2.02<br>110 K	11743	6.7 (7.0)	17.9<br>(11.4)	99.9<br>(100)	0.11<br>(0.14)	180.76	19.2	1.23	-2.0
5	2.71<br>110 K	11770	6.7 (7.0)	26.0<br>(16.9)	99.9<br>(100)	0.06<br>(0.09)	180.82	21.8	1.20	0.6
6	3.47<br>120 K	11769	6.7 (7.0)	26.7<br>(17.0)	99.9<br>(100)	0.06<br>(0.10)	180.86	22.2	1.25	1.0
7	4.2<br>130 K	11777	6.7 (7.0)	26.3<br>(16.4)	99.9<br>(100)	0.06<br>(0.10)	180.91	22.6	1.21	1.4
8	4.9<br>140 K	11790	6.7 (7.0)	25.5<br>(15.4)	99.9<br>(100)	0.06<br>(0.11)	180.97	23.1	1.19	2.0
9	7.59<br>150 K	11821	6.7 (7.0)	23.0<br>(12.4)	99.9<br>(100)	0.06<br>(0.14)	181.11	25.5	1.04	3.3
10	8.33<br>160 K	11854	6.7 (7.0)	21.8<br>(11.1)	99.9<br>(100)	0.07<br>(0.16)	181.29	26.9	0.96	5.7
11	9.06<br>140 K	n/a	n/a	n/a	n/a	n/a	181.21	n/a	n/a	n/a
12	9.80<br>120 K	11830	6.7 (7.0)	22.8<br>(10.4)	99.9<br>(100)	0.07<br>(0.17)	181.15	24.8	1.08	3.6

Table S9: Unit cell values for N9 neuraminidase variable and constant temperature series (N9TS and N9CS). Data were collected to 2.0 Å

DS	N9TS Temp	Unit cell Å	N9CS TempÅ	Unit cell Å
1	100 K	180.38	100 K	180.07
2	100 K	180.46	100 K	180.12
3	100 K	18055	100 K	180.22
4	100 K	180.59	100 K	180.23
5	110 K	180.64	100 K	180.25
6	110 K	180.71	100 K	180.33
7	120 K	180.78	100 K	180.39
8	130 K	180.96	100 K	180.38
9	140 K	180.98	100 K	180.49
10	150 K	181.02	100 K	180.33
11	160 K	181.12	100 K	180.44
12	165 K	181.11	100 K	180.52
13	100 K	180.72	100 K	180.40
14	100 K	180.80	100 K	180.51
15	100 K	180.95	100 K	180.61

Reference

Kmetko, J., Husseini, N. S., Naides, M., Kalinin, Y. & Thorne, R. E. (2006). *Acta Cryst. D* **62**, 1030-1038.
